# The leukemia inhibitory factor regulates fibroblast growth factor receptor 4 transcription in gastric cancer

**DOI:** 10.1007/s13402-023-00893-8

**Published:** 2023-11-09

**Authors:** Cristina Di Giorgio, Rachele Bellini, Antonio Lupia, Carmen Massa, Ginevra Urbani, Martina Bordoni, Silvia Marchianò, Rosalinda Rosselli, Rosa De Gregorio, Pasquale Rapacciuolo, Valentina Sepe, Elva Morretta, Maria Chiara Monti, Federica Moraca, Luigi Cari, Khan Rana Sami Ullah, Nicola Natalizi, Luigina Graziosi, Eleonora Distrutti, Michele Biagioli, Bruno Catalanotti, Annibale Donini, Angela Zampella, Stefano Fiorucci

**Affiliations:** 1 Department of Medicine and Surgery, University of Perugia, Perugia, Italy; 2 Department of Life and Environmental Sciences, University of Cagliari, Cagliari, Italy; 3 Net4Science Srl, University “Magna Græcia”, Campus Salvatore Venuta, Viale Europa, 88100 Catanzaro, Italy; 4 Department of Pharmacy, University of Naples Federico II, Naples, Italy; 5 Department of Pharmacy, University of Salerno, Salerno, Italy; 6 Azienda Ospedaliera Di Perugia, Perugia, Italy; 7 Department Surgical and Biomedical Sciences, University of Perugia Medical School, Perugia, Italy

**Keywords:** LIF, FGFR4, Gastric Cancer, Intestinal metaplasia, JAK1/STAT3 signaling pathway

## Abstract

**Purpose:**

The gastric adenocarcinoma (GC) represents the third cause of cancer-related mortality worldwide, and available therapeutic options remain sub-optimal. The Fibroblast growth factor receptors (FGFRs) are oncogenic transmembrane tyrosine kinase receptors. FGFR inhibitors have been approved for the treatment of various cancers and a STAT3-dependent regulation of FGFR4 has been documented in the *H.pylori* infected intestinal GC. Therefore, the modulation of FGFR4 might be useful for the treatment of GC.

**Methods:**

To investigate wich factors could modulate FGFR4 signalling in GC, we employed RNA-seq analysis on GC patients biopsies, human patients derived organoids (PDOs) and cancer cell lines.

**Results:**

We report that FGFR4 expression/function is regulated by the leukemia inhibitory factor (LIF) an IL-6 related oncogenic cytokine, in JAK1/STAT3 dependent manner. The transcriptomic analysis revealed a direct correlation between the expression of LIFR and FGFR4 in the tissue of an exploratory cohort of 31 GC and confirmed these findings by two external validation cohorts of GC. A LIFR inhibitor (LIR-201) abrogates STAT3 phosphorylation induced by LIF as well as recruitment of pSTAT3 to the promoter of FGFR4. Furthermore, inhibition of FGFR4 by roblitinib or siRNA abrogates STAT3 phosphorylation and oncogentic effects of LIF in GC cells, indicating that FGFR4 is a downstream target of LIF/LIFR complex. Treating cells with LIR-201 abrogates oncogenic potential of FGF19, the physiological ligand of FGFR4.

**Conclusions:**

Together these data unreveal a previously unregnized regulatory mechanism of FGFR4 by LIF/LIFR and demonstrate that LIF and FGF19 converge on the regulation of oncogenic STAT3 in GC cells.

**Graphical abstract:**

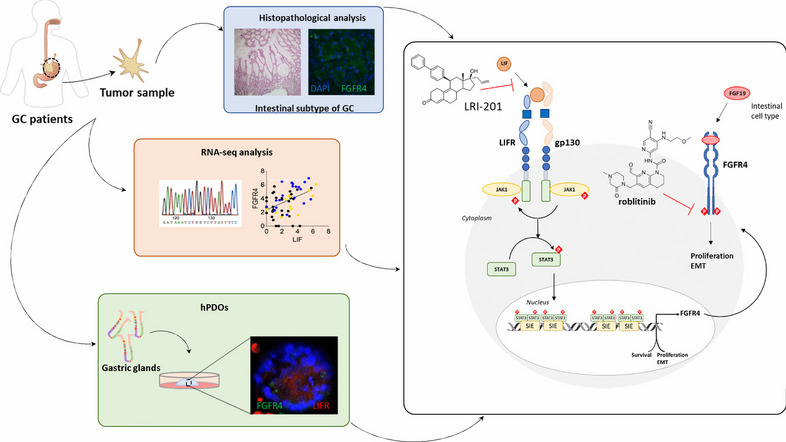

**Supplementary Information:**

The online version contains supplementary material available at 10.1007/s13402-023-00893-8.

## Introduction

Gastric cancer (GC) represents the fifth most commonly diagnosed malignancy but ranks third as leading cause of cancer-related mortality due to rapid progression, treatment resistance and an high metastasis rate. [1, 2]. GC is a heterogeneous disease with different histological phenotypes and genotypes. According to the Lauren classification, there are two major histological subsets: the intestinal type with well-to-moderately well-differentiated histology and the diffuse type with poorly differentiated histology; however, not all poorly differentiated GCs are of the diffuse type. The WHO classifies GCs into tubular, papillary, mucinous, mixed and poorly cohesive carcinomas [3, 4]. At the molecular level, four partially overlapping subtypes have been proposed by The Cancer Genome Atlas (TCGA) and by the Asian Cancer Research Group (ACRG) [5]. However, intra-patient and inter-patient heterogeneity impacts significantly on disease progression and poor response to chemotherapy, highlighting the need to identify novel, potentially druggable, targets.

Fibroblast growth factor receptors (FGFRs) are a group of four transmembrane tyrosine kinase receptors [6] that become phosphorylated/activated in response to binding by FGFs, a family of cell signaling proteins regulating a variety of biological functions including embryonic development, cell growth and differentiation, angiogenesis and cell migration [7]. Genetic alterations of FGFRs have been detected in several solid tumors, including intrahepatic cholangiocarcinoma [8, 9], endometrial cancer and GC [10, 11] and FGFRs inhibition has been recognized as an important therapeutic option for the treatment of FGFR-expressing tumors. As such several panFGFR, non-isoform selective inhibitors of FGFRs 1–4 [12], such as erdafitinib or pemigatinib [13] and futibatinib (TAS-120) [14], have been approved for clinical use [15]. Although the pan-FGFRs inhibitors have shown efficacy in FGFRs-driven tumors, their clinical benefit is limited by the development of resistance mutations and side effects, including FGFR4-mediated diarrhea [12]. FGFR4 is member of FGFR family with oncogenic potential in various solid neoplasms including, among others, stomach cancers [16]. In GC cells the Signal Transducer and Activator of Transcription (STAT)3 mediates the activation of FGFR4 caused by H.pylori proteins, suggesting that this pathway might have relevance in gastric oncogenesis[17]. STAT3 is a potent oncogenic factor acting on multiple regulatory pathways including AKT, MAPK and mTor, thus promotiong cell proliferation and de-differentiation [18]. Since, STAT3 is potential therapeutic target, we have settled up a study to clarify whether additional mechanisms regulate the STAT3/FGFR4 signaling in GC.

The Leukemia Inhibitory factor (LIF) is a regulatory factor belonging to IL-6 family of cytokines with a known oncogenic potential [19]. LIF acts on target cells by activating a heterodimeric cell membrane receptor (LIFR) made up by LIFRβ, a low-affinity subunit, and the glycoprotein (gp)-130, the signal transducer subunit. NGS studies have shown that LIF/LIFR are highly expressed in highly prevalent solid tumors, including stomach, pancreas, colon, liver and breast, and we and others have reported that high levels of LIF/LIFR associate with poor prognosis in GC patients and metastasis [20]. The downstream signaling of the LIF/LIFR pathway involves a Janus Kinase (JAK)-1-induced STAT3 phosphorylation [21]. Of relevance for the present study, STAT3 is over-phosphorylated in several LIF expressing GC [22].

Based on this background, we have settled a study to investigate whether a LIF/LIFR axis regulates the transcription of FGFR4 via STAT3 phosphorylation, leading to an aberrantly expression of FGFR4 especially in the histological intestinal subtype of GC. Moreover, we report the discovery of LRI-201, a novel LIFR inhibitor that negatively regulates FGFR4 in GC models. Targeting LIF/LIFR might avoid side effects linked to pan-FGFRs inhibitor and might be a promising therapeutic strategy for the treatment of cancers with aberrant FGFR4 activation.

## Material and methods

### Human Data

#### Patients

Paired GC samples were obtained from 31 patients undergoing surgical resection at the Surgery Unit of the Perugia University Hospital (Italy). Informed written consent was obtained from each patient before surgery. None of the patients had received chemotherapy or radiation before surgery. Specimen collection was carried out during surgery by a biologist and paired samples from non-neoplastic and neoplastic areas were collected. The samples were transported to the Gastroenterology laboratory in RNA later and then snap-frozen at—80 ºC until use.

#### The GSE66229 Dataset

The GSE66229 series from the ACRG (Asian Cancer Research Group) study, comprises 300 GC samples and 100 healthy tissue samples.

#### The Cancer Genome Atlas Stomach Adenocarcinoma (TCGA-STAD)

The TCGA-STAD, cohort comprises 350 GC samples and 31 healthy tissue samples.

### Transcriptome analysis

High-quality RNA was extracted from human non-neoplastic and neoplastic gastric mucosa using the PureLink™ RNA Mini Kit (Thermo Fisher Scientific), according to the manufacturer’s instructions. RNA quality and quantity were assessed with the Qubit® RNA HS Assay Kit and a Qubit 3.0 fluorometer followed by agarose gel electrophoresis. Libraries were generated using the Ion AmpliSeq™ Transcriptome Human Gene Expression Core Panel and Chef-Ready Kit (Thermo Fisher Scientific), according to the manufacturer’s instructions. Briefly, 10 ng of RNA was reverse transcribed with SuperScript™ Vilo™ cDNA Synthesis Kit (Thermo Fisher Scientific, Waltham, MA) before library preparation on the Ion Chef™ instrument (Thermo Fisher Scientific, Waltham, MA). The resulting cDNA was amplified to prepare barcoded libraries using the Ion Code™ PCR Plate, and the Ion AmpliSeq™ Transcriptome Human Gene Expression Core Panel (Thermo Fisher Scientific, Waltham, MA), Chef-Ready Kit, according to the manufacturer’s instructions. Barcoded libraries were combined to a final concentration of 100 pM, and used to prepare Template-Positive Ion Sphere™ (Thermo Fisher Scientific, Waltham, MA) Particles to load on Ion 540™ Chips, using the Ion 540™ Kit-Chef (Thermo Fisher Scientific, Waltham, MA). Sequencing was performed on an Ion S5™ Sequencer with Torrent Suite™ Software v6 (Thermo Fisher Scientific). The analyses were performed with a range of fold <  − 2 and >  + 2 and a p value < 0.05, using Transcriptome Analysis Console Software (version 4.0.2), certified for AmpliSeq analysis (Thermo-Fisher).

### Cell cultures

**2D CELL LINES.** Human gastric cell lines MKN74, MKN45, KATO III were obtained from the Japanase Collection of Research Bioresources, Human Science Resources Bank (Osaka, Japan). These cells were grown in RPMI 1640 medium (Sigma-Merk LIFe Science S.r.l. Milan, Italy) supplemented with 10% Fetal Bovine Serum (FBS), 1% L-Glutamine, 1% Penicillin/Streptomycin. HepG2, an immortalized human hepatocarcinoma cells line was grown in E-MEM Sigma-Merk LIFe Science S.r.l. Milan, Italy) supplemented with 10% Fetal Bovine Serum (FBS), 1% L-Glutamine, 1% Penicillin/Streptomycin. The cultures were maintained in a humidified 5% CO2 atmosphere, 37 °C. The cells, free from Mycoplasma contamination, confirmed by the use of Mycoplasma PCR Detection (Sigma) were regularly passaged to maintain exponential growth and used from early passages (< 10 passages after thawing). Before conducting the experiments, cells were plated, serum starved for 24 h and stimulated for 8–24-48 h or 72 h.

**3D CELL LINES.** Gastric glands were extracted from non-neoplastic and neoplastic mucosa excided from GC patients, and from the antrum of 4–8 weeks C57BL6/J mice. Human mucosa and murine stomach tissue was washed in cold PBS supplemented with antibiotics (Primocin, Invivogen). Then, tissue was cut in small fragments and incubated in Stripping buffer (HBSS with 10% FBS, 25 mM Hepes and 5 Mm EDTA) for 20 min at 37 °C with shaking. The fragments were subjected to enzymatic digestion by collagenase (1,5 mg/mL, Gibco) and hyaluronidase (20 µg/mL, Sigma) in 10 mL Advanced DMEM F12 (GIBCO) for 1 h at 37 °C with shaking. The gland suspensions were passed through 100 µM filter and washed twice in Advanced DMEM F12, seeded into Matrigel (50.000/50μL) and overlaid with Advanced DMEM F12 medium containing HEPES, Glutamax, Primocin, B27 (all from Invitrogen), n-Acetylcysteine 1 mM (Sigma-Aldrich), EGF 50 ng/mL (Invitrogen), R-spondin1 (200 ng/mL), Noggin (100 ng/mL), Wnt (100 ng/mL), FGF10 200 ng/mL (Peprotech), Gastrin 10 nM (Tocris), TGFβ-inhibitor 0.5 mM, RHOK-inhibitor 10 µM (Y-27632, Sigma-Aldrich) and LIF (10 ng/mL).

### Statistical analysis

**Fig. 1:**
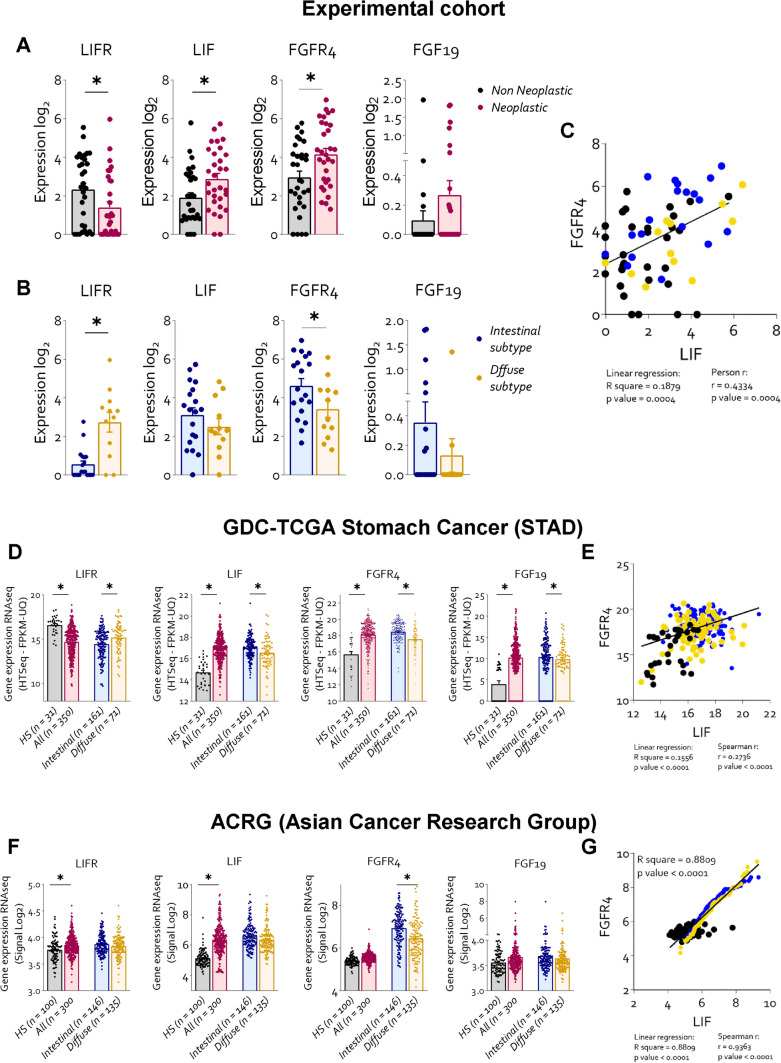
LIF and FGFR4 expression is upregulated in the histological intestinal subtype of GC. Transcriptome analysis of paired non neoplastic and neoplastic tissues in 31 patients with GC. Gene expression (Log2) of: LIFR, LIF, FGFR4 and FGF19 in **A)** non-neoplastic and neoplastic **B)** in intestinal and diffuse histological subtype of GC mucosa. **C)** Correlation graph between FGFR4 and LIF. RNA-seq analysis of healty, neoplastic mucosa, intestinal and diffuse histological type from TGCA-STAD repository and ACRG is also reported. **D)** Gene expression of: LIFR, LIF, FGFR4 and FGF19 from TGCA-STAD repository. **E)** Correlation graph between FGFR4 and LIF gene expression **F)** Gene expression of: LIFR, LIF, FGFR4 and FGF19 from ACRG repository. **G)** Correlation graph between FGFR4 and LIF. Each dot represents a patient. Results are the mean ± SEM. * p < 0.05

We first performed the Kolmogorov–Smirnov test for accessed whether our data are in a normal distribution. The Student t test was performed on experimental set composed by two groups: Welch’s correction for samples with Gaussian distribution and Mann Whitney test for data without a Gaussian distribution. The one-way ANOVA were used for statistical comparisons on experimental set with more of two groups: Brown-Forsythe & Welch for samples with Gaussian distribution and Kruskal–Wallis test for data without a Gaussian distribution. For the correlation studies in Fig. 1 the correlation was calculated with Pearson r for data with Gaussian distribution and with Spearman r for data that did not have a Gaussian distribution. The straight line in the graphs was instead calculated by linear regression. All test was carried out using the Prism 8.0 software (GraphPad) (*p < 0.05).

## Results

### FGFR4 is upregulated in human gastric cancers

This study involved the RNA transcription profile of paired gastric mucosa obtained from 31 GC patients, who underwent surgery at the Perugia University Hospital between 2013–2018. The histological classification according to the Lauren criteria was as following: 19 patients had the intestinal subtype and 12 the diffuse subtype of GC. The trascriptome analysis of paired GC samples (Fig. 1 A and Supplementary Fig. 1) revealed that, in comparison to non-neopastic pairs, the expression of LIF and FGFR4 mRNAs was significantly increased in GC pairs and that for both factors the higher expression occurred in the intestinal subtype (Fig. 1 B). In contrast, the other three members of FGFR family showed no differential expression between neoplastic and non-neoplastic tissues (Supplementary Fig. 1). These data were confirmed by the immunofluorescence analysis (IF) of paired GC biopsies. Indeed, as shown in Supplementary Fig. 2 A higher levels of FGFR4 protein were detected in the intestinal subtype of with the neoplastic pairs showing the higher levels of protein in comparison to the non-neoplastic mucosa and the diffuse subtype of GC samples. Furthermore, a regression analysis carried out in all 31 pairs (neoplastic and non-neoplastic), demonstrated a positive correlation between the expression of FGFR4 and LIF mRNA levels (Fig. 1I) (LIF/FGFR4: p value < 0.0001; R square = 0.1879), suggesting a potential positive interaction between these two factors.

To clarify whether the differential expression of LIF and FGFR4 cross the various GC subtypes translate in to a the prognostic prediction we have carried out a Kaplan Meier analysis [23] of patients survival rate after their partition into LIF-high and LIF-low and FGFR4-high and FGFR4-low groups according to different levels of expression of LIF and FGFR4 (Supplementary Fig. 2B-C). The Kaplan–Meier plotter survival analysis indicated that, despite higher levels of expression of the receptor occurred in the intestinal subtype, high FGFR4 levels are a poor prognostic indicator in patients with diffuse type of GC, but not in patients with intestinal subtype. The results obtained in our cohort were validated by two external cohorts: The Cancer Genome Atlas Stomach Adenocarcinoma (TCGA-STAD) (Fig. 1K-O) and the Asian cancer research group (ACRG) databases (Fig. 1P-T). The analysis of these two cohorts confirmed that LIF and FGFR4 were among the most upregulated gene in the intestinal subtype of GC (Fig. 1 L-M and Q-R) compared to healthy stomach mucosa. Moreover, in both cohorts the expression of LIF correlated significantly with the expression of FGFR4 (p < 0.05) (Fig. 1 O and T), while the Kaplan–Meier plotter survival analysis of ACRG repository confirmed the results of our cohort of patients, showing that high levels of FGFR4 correlated with a poor survival in the diffuse sub-type of GC (Supplementary Fig. 3 K).

### LIFR modulation regulates FGFR4 expression

Because the LIF/LIFR complex promotes an oncogenic development in several cancers [24–26], and the expression of LIF mRNA correlates with expression of FGFR4 in GC, then we saought to investigate whether the LIF regulates the expression of the FGFR4 in gastric adenocarcinoma cell lines. In a preliminary analysis of LIF, LIFR and FGFR4 mRNA expression in various GC cell lines, MKN45, MKN74, and KATO III, we have found that MKN45 cells exhibited the higher levels of three genes (Supplementary Fig. 4) and were, therefore, selected for the following in vitro investigations.

**Fig. 2:**
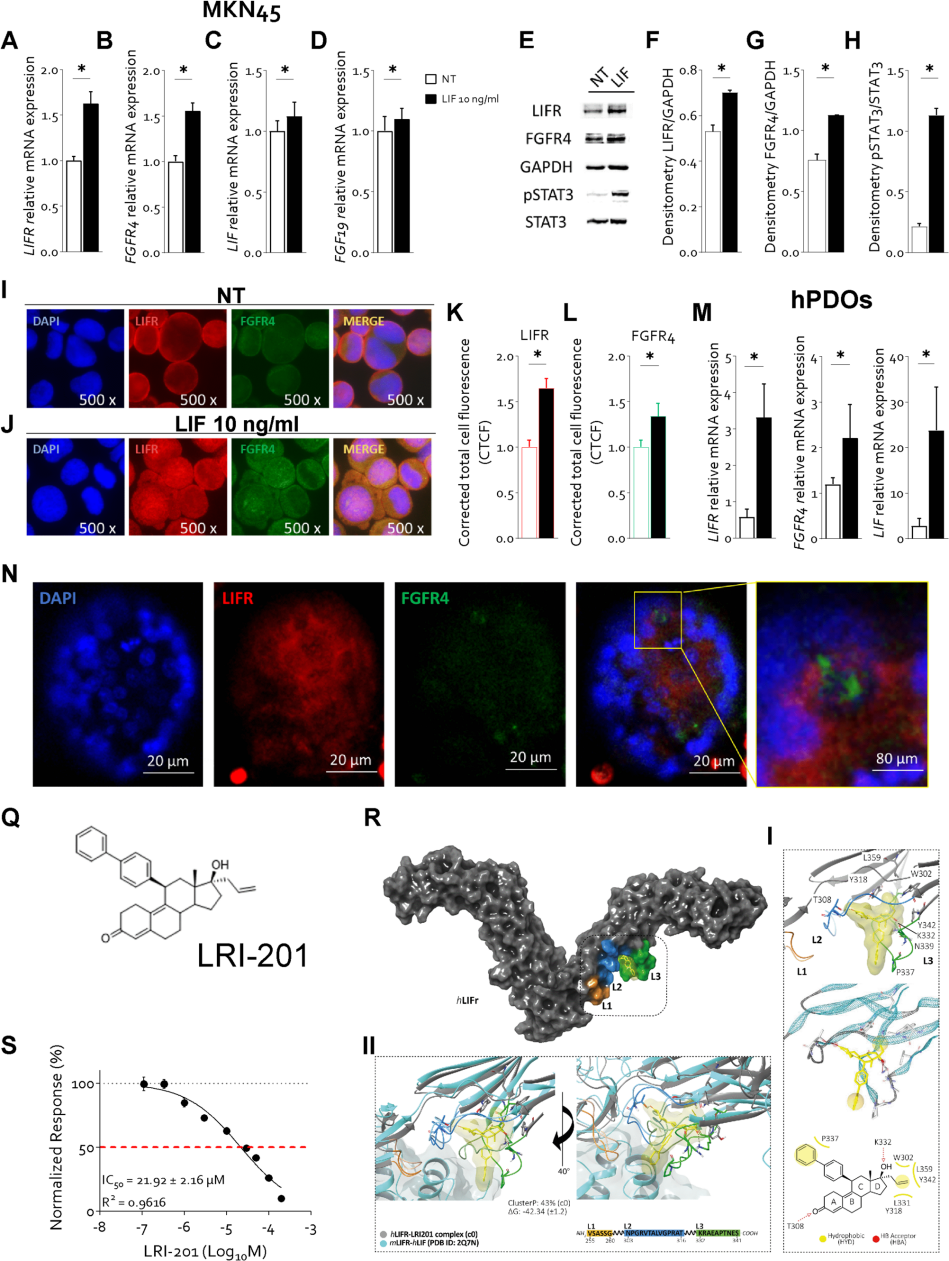
**LIF increases the expression of FGFR4 receptor in a STAT3 dependent manner in 2D and 3D-GC coltures. A-L) MNK45 cell lines was exposed to LIF for 24 h or left untreated.** Relative mRNA expression of **A)** LIFR, **B)** FGFR4, **C)** LIF and **D)** FGF19. Each value is normalized to GAPDH and is expressed relative to not treated group, which are arbitrarily set to 1. **E)** Representative Western blot analysis of phospho-STAT3 and STAT3 proteins. Densitometric analysis demonstrating **F)** LIFR/GAPDH, **G)** FGFR4/GAPDH and **H)** phospho-STAT3/STAT3 ratio. IF analysis (Magnification 500x) of LIFR (red) and FGFR4 (green) in **I)** left untreated cells and cells exposed to **J)** LIF (10 ng/mL). The estimated corrected total cell fluorescence (CTCF) of **K)** LIFR and **L)** FGFR4. **M–N) hPDOs was established from GC patient mucosa, they were exposed to LIF or left untreated for 1 week. M)** Relative mRNA expression ofLIFR, FGFR4 and LIF. **N)** IF analysis of LIFR (red) and FGFR4 (green) on PDOs. Results are the mean ± SEM of 3–5 samples for group. (*represents statistical significance versus NT, p < 0.05. **Q)** 2D structure of the novel LIFR antagonist, LRI-201. **R) The hLIFR-LRI-201 complex**. The hLIFR-LRI-201 complex. **I)** The zoom-view of the hLIFR-LRI-201-c0 cluster obtained after 200 ns of MD (gray) and **II)** superimposed respect to the hLIF-mLIFR complex (PDB: 2Q7N) (cyan). The principal residues are labelled and highlighted in stick, while both hydrophobic (HYD) and H-Bond Acceptor (HBA) pharmacophore features are colored in yellow and red, respectively. All H-bonds are highlighted in dashed lines. The pocket is defined by three loops, namely L1 (255-VSASSG-260), L2 (303-NPGRVTALVGPRAT-316), and L3 (332-KRAEAPTNES-341). **S)** LRI-201 inhibition activity of LIFR/LIF binding accessed by a cell-free AlphaScreen assay

To examine the potential of the LIF/LIFR patways in regulating the FGFR4, we have first exposed MKN45 to 10 ng/mL LIF (see ref. [20] for details) and found that this cytokine strongly induces the expression of LIFR (Fig. 2 A) and FGFR4 (Fig. 2 B), mRNAs and proteins (Fig. 2 F and G). Exposure of MKN45 cells to LIF also lead to the activation of the STAT3 pathway as shown by enhanced expression of LIFR and FGFR4 and STAT3 phosphorylation on Tyr705 (Fig. 2 E–H). The IF analysis confirmed a robust induction of LIFR and FGFR4 and their co-localization at the cell membrane in response to exposure MKN45 cells to LIF (Fig. 2 I-L).

To confirm these data at translational level, we have established human patient-derived organoids (hPDOs) from GC patients using non-neoplastic and neoplastic cancer pairs as described above. Challenging hPDOs with 10 ng/ml LIF resulted in potent induction of the expression of LIFR, FGFR4 and LIF mRNAs in non neoplastic hPDOs (only FGFR4 not shown) and in neoplastic organoids (Fig. 2 M). These findings are specie-specific since LIF failed to regulate FGFR4 in gastric murine organoids (Supplementary Fig. 8). These results were confirmed by IF analysis as shsown in Fig. 2 N.

Because regulation of FGFR4 by LIFR has a therapeutic potential, and there are no clinically approved LIFR antagonists, we building on the recept repositioning of mifespristone as LIFR antagonist [22] we have developed a novel small molecule LIFR antagonist, LIFR-inhibitor (LRI)-201 (Supplementary Results) and tested it in vitro and in silico models, as described previously [27]. The molecular dynamic demonstrated that, despite the high flexibility of the loops surrounding the ligand binding site, the binding mode of LRI-201 is stable within such binding pocket formed by loops L2 and L3, leading to the formation of a hydrogen bond (H-bond) between the carbonyl group in position 3 and Thr308 tethering the ligand to the L2 loop, the ring D to the L3 loop, through interactions of the allyl double bond with Tyr318, Leu331 Tyr342,Leu359 and Trp302, (Fig. 2R-I; Supplementary Figure S6), while the biphenyl ring protruded toward the LIF binding interface establishing hydrophobic interaction with Pro337 (Fig. 2 R-I). According with the proposed binding mode, LRI-201 sensibly altered the structure of the LIF binding site by widening the distance between L2 and L3 especially if compared to the hLIF-mLIFR architecture (Fig. 2 R-II). Confirming these computational studies LRI-201 effectively inhibits LIF/LIFR interaction with an IC50 of 21.92 ± 2.16 µM in the Alpha Screen assay (Fig. 2 S).

**Fig. 3:**
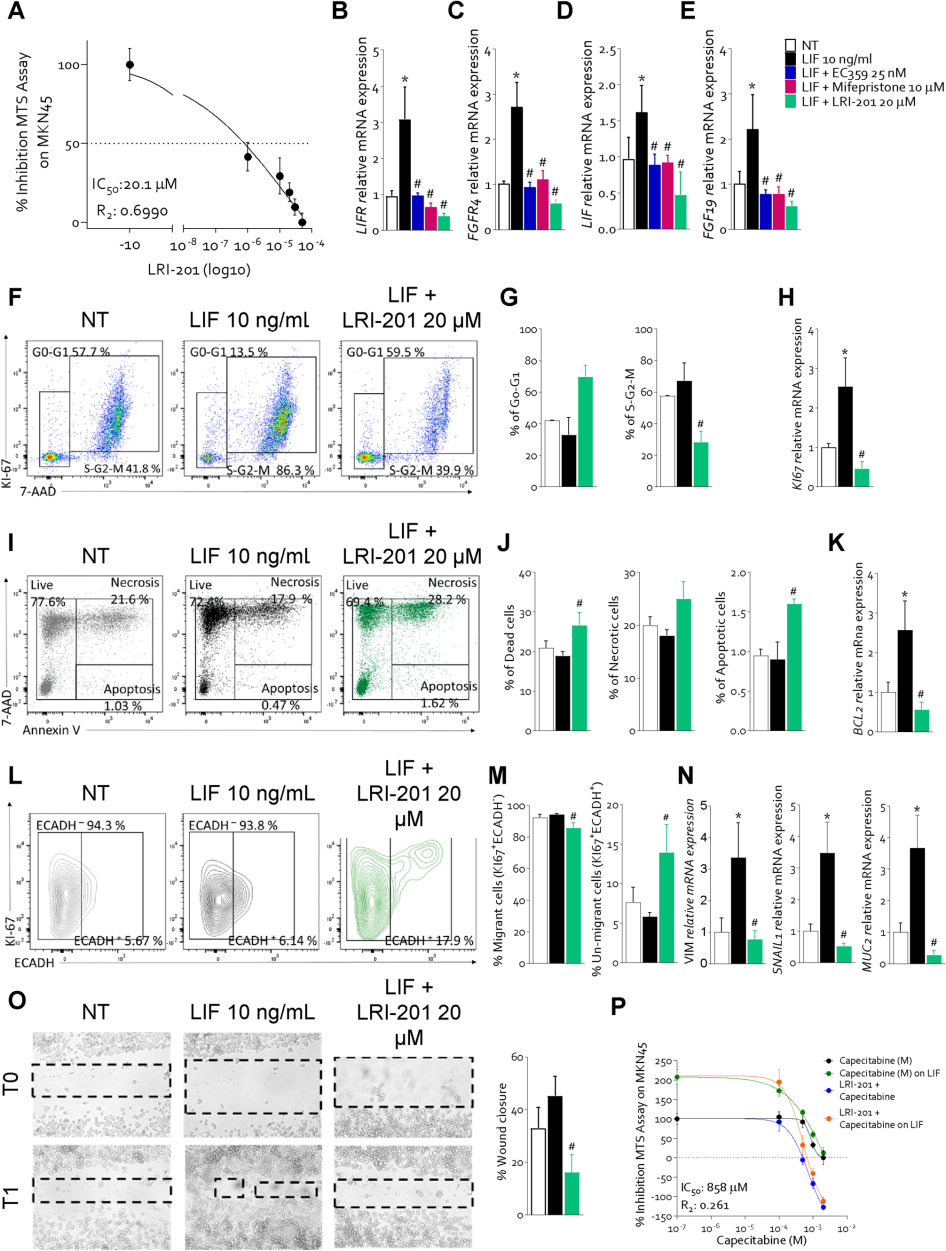
**LRI-201 reverted LIF-Induced proliferation and EMT process in MKN45 cells. A)** Dose–response curve of LRI-201 (0.1, 1, 10, 20, 30 and 50 µM) was determined using MTS assay. Relative mRNA expression of **B)** LIFR, **C)** FGFR4, **D)** LIF and **E)** FGF19 in MKN45 cells left untreated or exposed to LIF (10 ng/mL) alone or plus LIFR inhibitors (25 nM of EC359, 10 μM of mifepristone and 20 μM of LRI-201). Each value is normalized to GAPDH and is expressed relative to those of NT, which are arbitrarily set to 1. Cell cycle phase analysis was performed by Ki-67/7-AAD staining through IC-FCM. **F)** Representative IC-FCM shows cell cycle fraction in NT, LIF 10 ng/mL and LIF + LRI-201 20 µM groups. Frequencies of cells in the **G)** G0-G1 phase and S-G2-M phase. **H)** Relative mRNA expression of Ki-67 in each experimental group. **I)** Representative IC-FCM shows Annexin V^+^ cells in each experimental group. Data shown are frequencies of **J)** Dead single cells, Necrotic single cells and Apoptotic single cells. Relative mRNA expression of **K)** BCL2 in each group. **L)** Representative IC-FACS of Ki-67^+^ cells and relative percentage of **M)** ECADH^−^ and ECADH^+^ cells. Relative mRNA expression of EMT markers **N)** VIM, SNAIL1 and the intestinal marker, MUC2. **O)** A scratch wound healing assay is shown. MKN45 cell monolayers were scraped in a straight line using a p200 pipette tip, then they are left untreated or primed with LIF 10 ng/mL alone or in combination with LRI-201 20 µM. The wound generated was captured at 0 h and 24h of incubation with the compounds above described. The images show cell migration at the two times point indicated and percentage of wound closure at 24 h. **P)** Dose–response curve of capecitabine alone or in combination with LIF and/or LRI-201 was determined using MTS assay. Results are the mean ± SEM of 3–5 samples for group. (*represents statistical significance versus NT, # versus LIF p < 0.05.)

Exploiting the LRI-201, then, we have investigated the impact of LIFR inhibition on GC cells growth and proliferation. For this purpose, MKN45 were grown in a serum free medium, and exposed to 10 ng/mL LIF alone or in combination with increasing concentrations of LRI-201 (1, 10, 20, 30, 50 µM) for 24 h. As shown in Fig. 3 A, LRI-201 reversed the LIF-proliferative effect of LIF in a concentration-dependent manner. Additionally, LIFR inhibition by LRI-201 reversed the induction of LIFR, FGFR4, LIF and FGF19 mRNAs caused by LIF. Similar data were obtained using other LIFR inhibitors: EC359 and mifepristone (Fig. 3 B-E). The effect of LRI-201 on cell replication was also assessed using intracellular flow cytometry (IC-FCM) in MKN45 cells stained with Ki-67 and 7-AAD (Fig. 3 F). As shown in Fig. 3F-G, while exposure of MKN45 cells to LIF promoted the replicative S-G2-M transition, this effect was significantly reversed by LRI-201 (20 µM), that also reduced the expression of KI67 gene (Fig. 3 H). LRI-201 treatment also increased the percentage of Annexin V^+^ cells (p < 0.05), as determined by Annexin V staining (Fig. 3 I-J) and led to a reduction of the expression of the anti-apoptotic BCL2 gene (Fig. 3 K).

LIFR inhibition also prevents the acquisition of invasive phenotype promoted by challenging GC cells with LIF [28]. Indeed, as shown in Fig. 3 L-M, while exposure of MKN45 cells to LIF increased the rate of the un-migrant E-cadherin^+^ cells, as determined by Ki-67/E-cadherin IC-FACS staining and reversed the induction of the EMT markers such as vimentin and SNAIL1 (Fig. 3 N), caused by LIF. In addition, LRI-201 treatment also reversed the increment of MUC2 mRNA expression (Fig. 3 N) induced by LIF suggesting a potential role of the axis LIF/LIFR/FGFR4 in the onset of cancer. Furthermore, exposure to LRI-201 reversed the acquisition of a migratory phenotype caused by LIF, as assessed in the scratch wound healing assay (Fig. 3 O). Thus, while LIF promoted the cell migration and wound closure, with a reduction of the wound area by ≈45% at 24 h, the treatment with LRI-201 reduced MKN45 migration and the wound closure of ≈ 16%, similarly it was observed at 48 h (not shown).

Furthermore, considering that LIF overexpression is associated with resistance to chemotherapy [29], we have examined whether exposure to LRI-201 improves resistance of MKN45 cells to capecitabine [30]. For this purposes MKN45 were exposed to LIF alone or in combination with increasing concentrations of capecitabine (0.1,0.5, 1, 2 mM), and LRI-201 (20 µM). The results of these studies (Fig. 3 P) demonstrated that while exposure to LIF promoted capecitabine resistance and increased the proliferation rate, these effects were reversed by LRI-201. Taken together, these findings suggest that LIFR antagonism in GC cell lines reverses the positive modulation of FGFR4, acquisition of an iper-proliferiferative, migratory phenotype and chemoresistance induced by LIF.

### FGFR4 expression is induced via direct binding of STAT3 on FGFR4 promoter

**Fig. 4:**
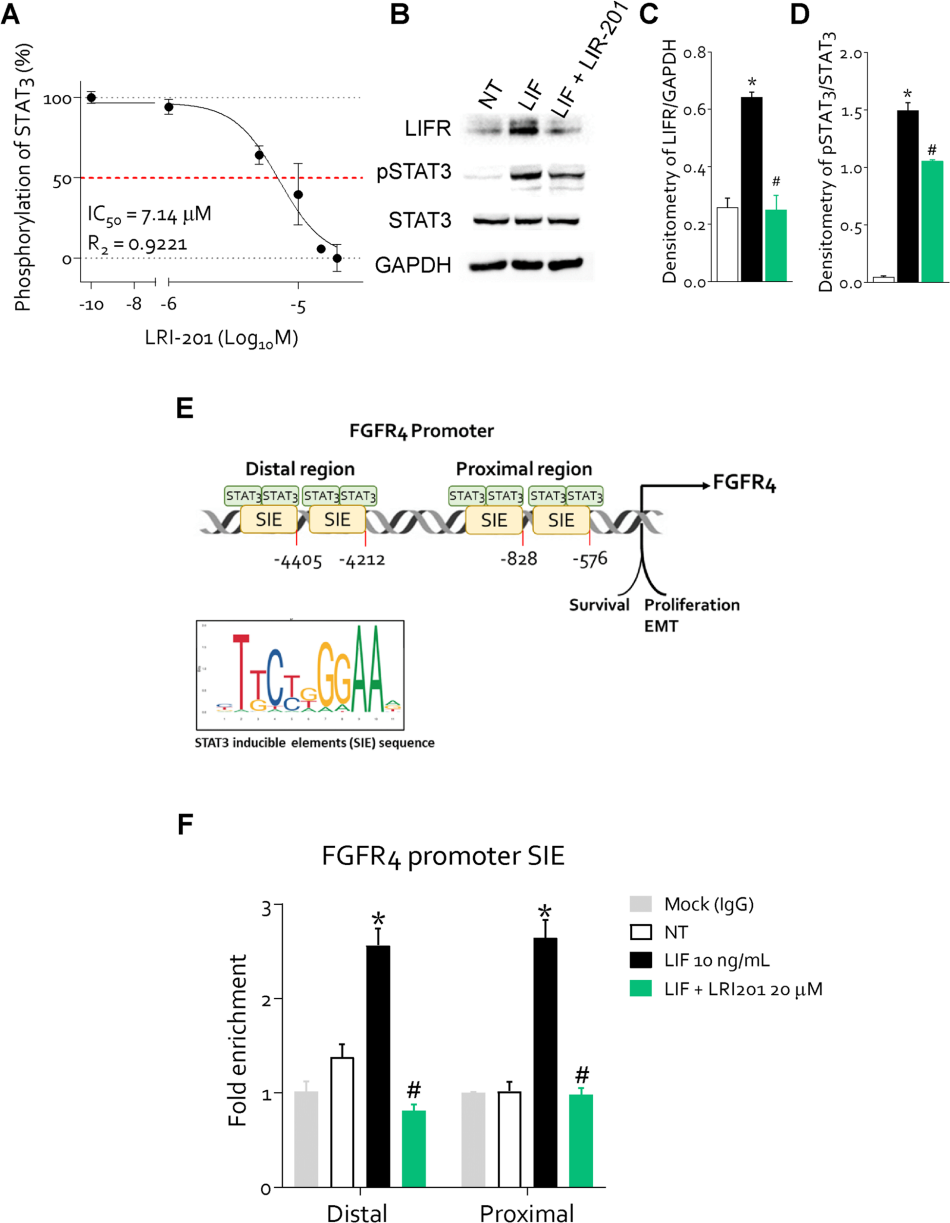
**FGFR4 expression is induced by LIF via direct binding of STAT3 on the FGFR4 promoter. A)** STAT3 transactivation on HepG2 cells. **B)** Western blot analysis of LIFR phospho-STAT3 and STAT3 proteins in MKN45 cells NT or exposed to LIF (10 ng/mL) alone or in combination with LRI-201 (20 µM). Densitometric analysis demonstrating **C)** phospho-STAT3/STAT3 ratio and **D)** LIFR/GAPDH **E)** A schematic diagram shows the location of STAT3 putative binding regions on the FGFR4 promoter. UTR, untranslated region. **F)** ChIP assay: cell lysates of MKN45 were immunoprecipitated with anti-STAT3 or control IgG (Mock IgG), and the presence of specific regions of FGFR4 promoter in the immunoprecipitates was determined by real-time PCR. Results are the mean ± SEM of 3–5 samples for group. (*represents statistical significance versus NT, and # versus LIF, p < 0.05)

To further investigate the mechanism involved in STAT3 regulation of FGFR4, we have performed a transactivation assay in HepG2 cells transiently transfected with a reporter plasmid containing STAT3 inducibile elements (SIE) plasmide cloned upstream to the luciferase gene, as described previously [27]. Transfected cells were then challenged with the LIF protein alone or in combination with the LIFR inhibitor LRI-201. As shown in Fig. 4 A, while exposure of HepG2 transfected cells to LIF activates a STAT3-dependent luciferase transcription, this pattern was abrogated by co-incubating the cells with the LIFR antagonist, with an IC50 of 7.14 µM. These results were confirmed by Western blot analysis. As shown in Fig. 4 B-D, LRI-201 reversed the induction of LIFR and attenuated STAT3 phosphorylation induced by LIF. Since this data suggest that LIF-induced STAT3 is involved in the transcriptional regulation of FGFR4, we have carried out a promotorial analysis of the FGFR4. The results of this analysis allowed the identification of four putative STAT3 inducing elements (SIE) in the FGFR4 promoter region. Two site were located in the proximal promoter, respectively -576 bp and—828 bp from the ATG starting sequence, while other two sites were detected in the distal promoter of FGFR4, at -4212 bp and—4405 bp from the ATG starting sequence (Fig. 4E). To confirm the functional relevance of these SIE on the FGFR4 promoter regions, we carried out a chromatin immunoprecipitation (ChIP) using one pairs of primers (P1) designed to cover the SIE in the distal binding sites and one pairs of primers (P2) for the proximal binding sites on FGFR4 promoter. Real-time analysis using STAT3-conjugated DNA samples showed amplification with bothe the P1 and P2 primers, suggesting that STAT3 binds all these candidate binding sites. The ChIP assay demonstrated that exposure to LIF induced a significant increase in the amplification of the fragments containing P1 and P2 binding sites, compared to cells exposed to LIF plus LRI-201. These results indicated that LIF induced FGFR4 expression is mediated by STAT3 binding to SIE in the FGFR4 promoter (Fig. 4F).

### FGFR4-inhibition mitigates the pro-oncogenic effects of LIF in MKN45 cells

**Fig. 5:**
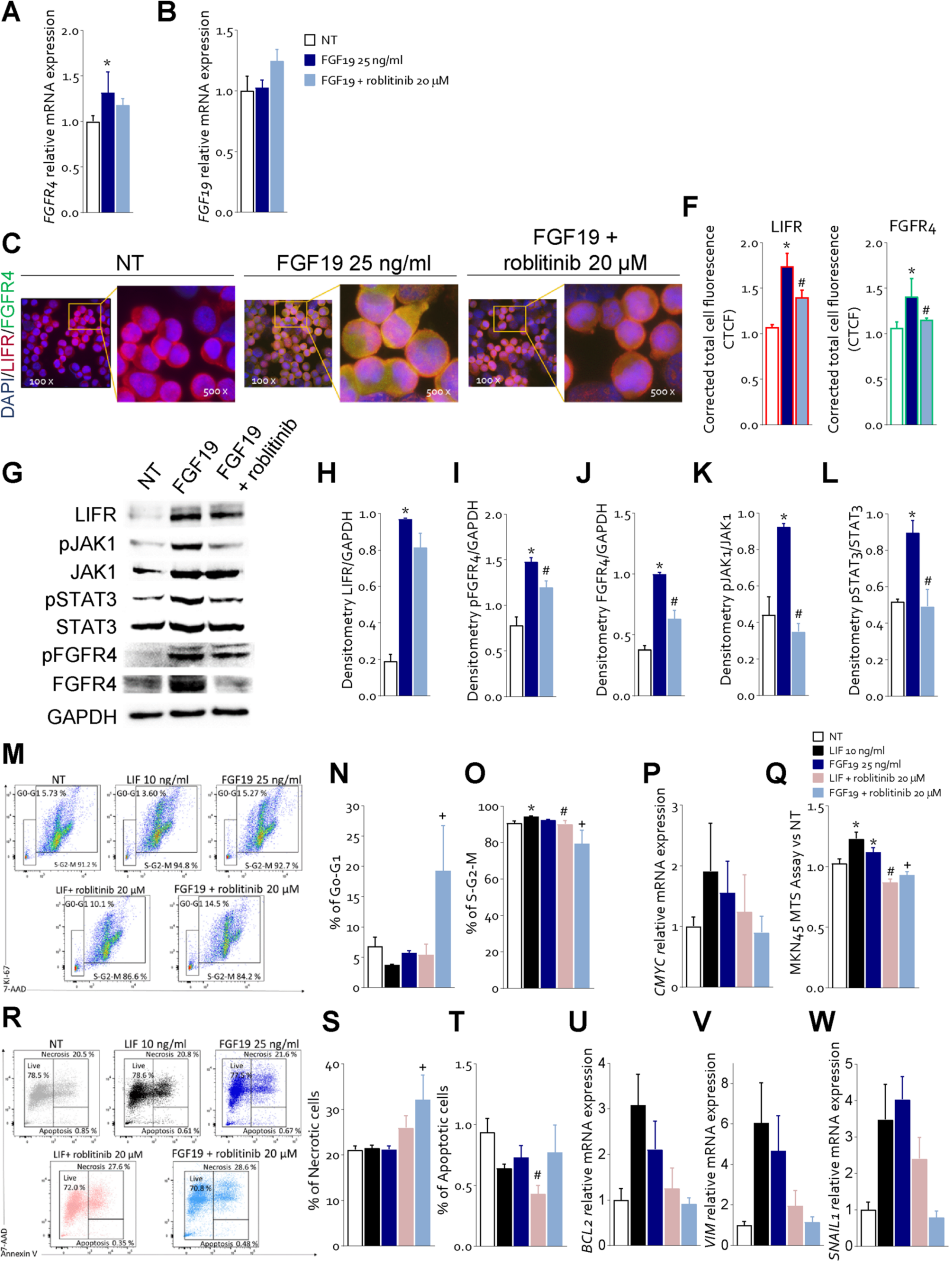
**FGFR4 inhibition with roblitinib mitigates LIF pro-oncogenic effects. A-L)** In the first experimental set MKN45 cells were left untreated or exposed to 25 ng/mL of FGF19 alone or in combination with 20 µM of roblitinib. Relative mRNA expression of **A)** FGFR4 and **B)** FGF19. Each value is normalized to GAPDH and is expressed relative to those of NT, which are arbitrarily set to 1. **C -E)** IF staining (Magnification 100 × on the left, 500 × on the right) of LIFR (red) and FGFR4 (green) in each experimental group. **F)** Estimated corrected total cell fluorescence (CTCF) of LIFR and FGFR4. **G)** Representative Western blot analysis of the pathway-associated LIFR, phospho-JAK1, JAK1, phospho-FGFR4, FGFR4, phospho-STAT3 and STAT3 proteins. Densitometric analysis of **H)** LIFR/GAPDH, **I)** phospho-FGFR4/GAPDH, **J)** FGFR4/GAPDH, **K)** phospho-JAK1/JAK1 ratio and **L)** phospho-STAT3/STAT3 ratio. In the second experimental set MKN45 cells were left untreated or exposed to LIF (10 ng/mL), FGF19 (25 ng/mL), both alone or in combination with of roblitinib (20 µM). Cell cycle phase analysis was performed by Ki-67/7-AAD staining through IC-FCM. **M)** Representative IC-FCM shows cell cycle fraction in each experimental group. Frequencies of cells in the **N)** G0-G1 phase and **O)** S-G2-M phase in each experimental group. Relative mRNA expression of **P)** c-MYC in each experimental group. **Q)** MTS assay **R)** Representative IC-FCM shows Annexin V^+^ cells. Data shown are frequencies of **S)** Necrotic single cell and **T)** Apoptotic single cell. Relative mRNA expression of **U)** BCL2, **V)** VIM and **W)** SNAIL1. Results are the mean ± SEM of 3–5 samples for group. (*represents statistical significance versus NT, and # versus LIF, + versus FGF19 p < 0.05)

**Fig. 6:**
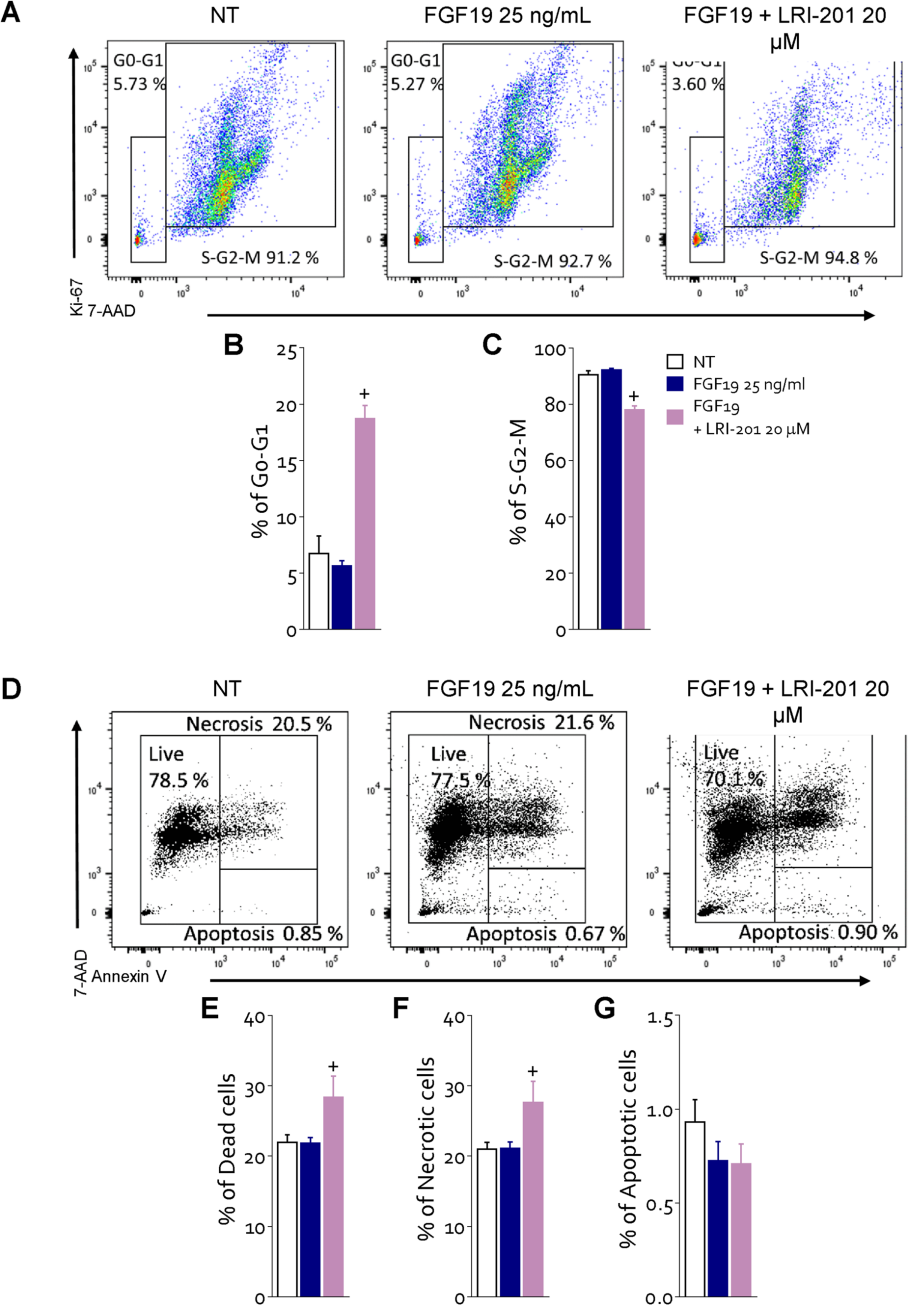
**LRI-201 mitigates FGF19 proliferative and anti-apoptotic effects A)** Representative IC-FACS shows cell cycle fraction in NT, FGF19 25 ng/mL and FGF19 + LRI-201 20 µM groups. Frequencies of cells in the **B)** G0-G1 phase and **C)** S-G2-M phase. Results are the mean ± SEM of five samples for group. **D)** Representative IC-FACS shows Annexin V^+^ cells in each experimental group. Data shown are frequencies of **E)** Dead single cells, **F)** Necrotic single cells and **G)** Apoptotic single cells. Results are the mean ± SEM of five samples for group. (*represents statistical significance versus NT, and # FGF19 p < 0.05)

**Fig. 7:**
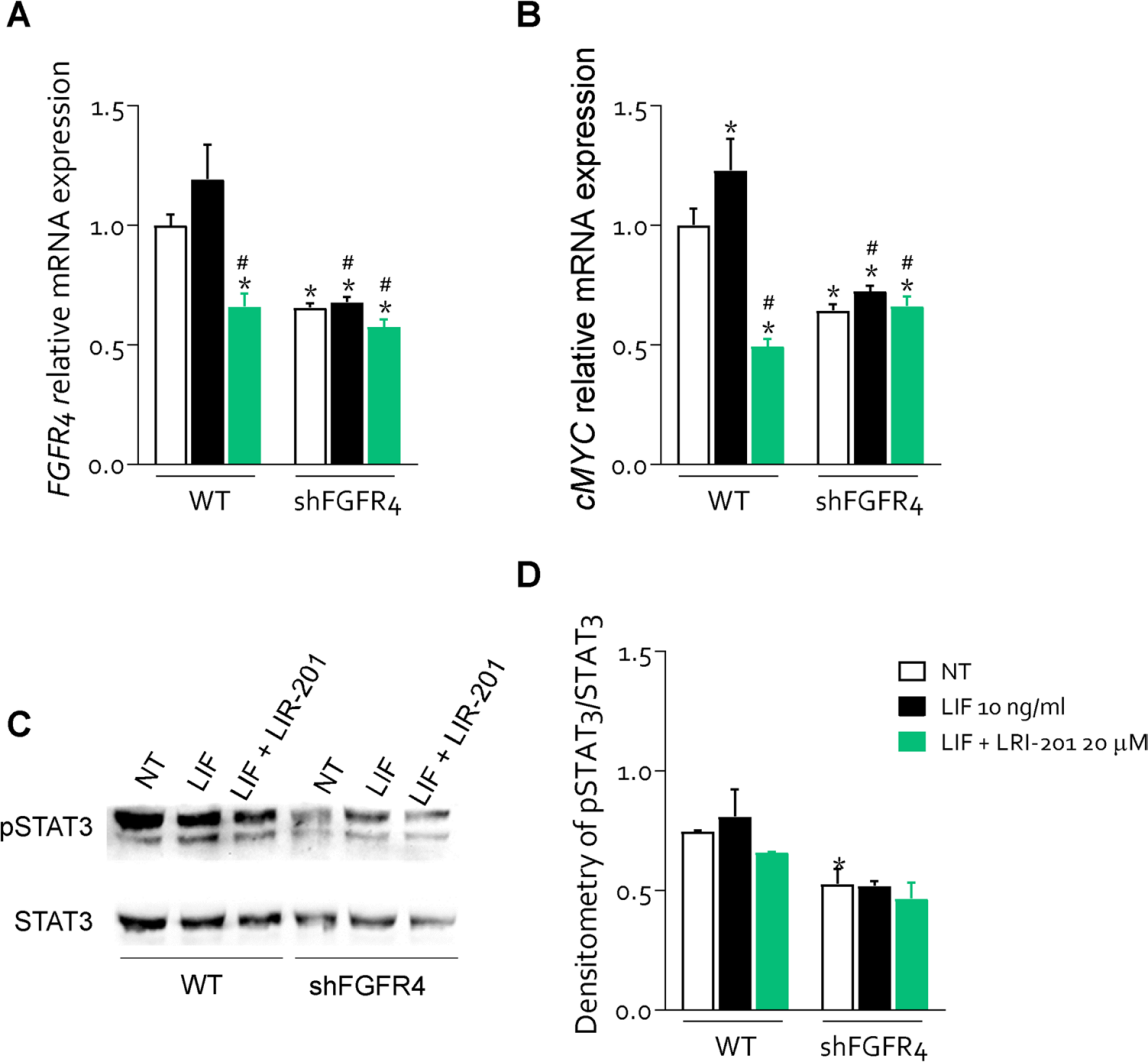
**FGFR4 silencing reduced LIF pro-oncogenic effects.** MKN45 was transfected with an anti-FGFR4 siRNA, then cultured in a medium with LIF (10 ng/mL) alone or in combination with LRI-201 (20 µM) and compared to MKN45 untrasfected. Relative mRNA expression of **A)** FGFR4 and **B)** cMYC in Each value is normalized to GAPDH and is expressed relative to those of NT, which are arbitrarily set to 1. **C**) Western blot analysis of LIFR phospho-STAT3 and STAT3 proteins in both Densitometric analysis demonstrating **D**) phospho-STAT3/STAT3 ratio. Results are the mean ± SEM of 3–5 samples for group. (*represents statistical significance versus NT, and # versus LIF, p < 0.05)

Since FGF19 is the endogenous ligand of FGFR4 and is expressed in GC (Fig. 1), we have then investigated whether activation of FGFR4 by FGF19 reproduces the same functional effects of LIF and whether inhibition of FGFR4 kinase domain by roblitinib [31] mitigate the effects of FGF19 and LIF on GC cells (Fig. 5). For these purposes MKN45 cells were growth with 25 ng/mL of FGF19 alone or in combination with roblitinib (20 μM). Exposure of MKN45 cells to FGF19 increased the expression of FGFR4 and FGF19 mRNA (Fig. 5 A-B) and protein, as assessed by IF (Fig. 5 C-F) and Western blot analyses (Fig. 5 G). Surprisingly, the results of these experiments demonstrated that exposure of MKN45 cells to FGF19 significantly increased the expression of LIFR by approximately two-fold (Fig. 5G and H) and this effect was only partially reduced by roblitinib. Moreover, FGF19 promoted FGFR4, JAK1 and STAT3 phosphorylation (Fig. 5G-L) and these effects were reversed by FGFR4 inhibition with roblitinib. Since these findings indicate a reciprocal regulation of the LIFR and FGFR4 pathways we have then investigated whether inhibition of FGFR4 by roblitinib reverses the effects of LIF, in addition of FGF19 on MKN45 cells. As shown in Fig. 5M-O, exposure to roblitinib reduced the percent of replicative cells, cMYC mRNA expression (Fig. 5P) and proliferation rate induced by LIF and FGF19 (Fig. 5Q). Moreover, roblitinib increased the apoptotic cell rate (Fig. 5R) and BCL2 mRNA expression (Fig. 5U) and downregulated the vimentin and SNAIL1 mRNA expression induced by LIF and FGF19 (Fig. 5 V and W). Further supporting LIF/FGF19 overlapping, LRI-201 reverted the effects exerted by FGF19 on cell cycle: increasing the rate of G0-G1 (Fig. 6 B) cells and reducing the rate in replicative cells (Fig. 6 C) and increasing the death cell rate (Fig. 6 E). Finally, FGFR4 knockdown performed in MKN45 by transfecteing cells with an anti-FGFR4 siRNA, abrogated the pro-oncogenic effect of LIF. As illustrated in Fig. 7, although the knockdown resulted in a reduction of ≈30% of FGFR4 mRNA expression, shFGFR4 MKN45 exposed to 10 ng/mL of LIF showed decreased expression levels cMYC mRNA and the treatment with LRI-201 was unable to revert this pattern. Collectively, these findings confirm a functional overlap between the LIF/LIFR/STAT3 and FGF19/FGFR4 pathway in promoting cell survival and EMT in GC.

## Discussion

Here we report that activation of LIF/LIFR/STAT3 pathway regulates the expression and oncogenic activities of FGFR4 in GC. LIF is a highly pleiotropic member of the IL-6 family of cytokines, upon binding to the LIFR complex promotes the crosstalk between the extracellular matrix (ECM) and cancer cells and mediates the transition through a pro-invasive phenotype of stromal fibroblasts and cancer cells [32]. Despite several tumors exhibit upregulated LIF/LIFR-STAT3 signaling via autocrine and paracrine mechanisms, the mechanistic relevance of this pathway and its therapeutic potential in GC remains elusive. In several cancer subsets, induction of oncogenic STAT3 [33] associates with increased expression of FGFR4 [34], a member of a tyrosine kinase family of FGFRs involved in cell growth, differentiation and migration. Since an increased expression and FGFR4 in the tumor tissues predicts a poor prognosis in various cancers and its targeting has proven promising in the treatment of FGFR4-mediated tumors, understanding molecular mechanisms that regulate FGFR4 expression in GC might be of therapeutic relevance.

In the present study, we report that the transcriptome analysis of cancer tissues from a cohort of locally advanced GCs express a dysregulated expression of LIF/LIFR and FGFR4. Results of RNAseq analysis presented in Fig. 1, highlighted the concept that FGFR4 was the most significantly up-regulated gene among FGFRs, and its expression is strictly correlated with the expression of pro-oncogenic cytokine, LIF. Furthermore, based on Lauren classification, high levels of LIF and FGFR4 were found to be predominant in the intestinal subtype although their higher expression was not correlated with reduced patient survival. In contrast, among the patients with the Lauren’s diffuse histotype, high levels of FGFR4 were associated with a worst prognosis.

To validate these findings, we have employed a bioinformatic approach and examined two external repositories of human GC, the STAD-TGCA and ACRG databases. The analysis of these two additional cohorts were largely consistent with the fact that FGFR4 was overexpressed in the Lauren’s intestinal subtype of GC, and its expression significantly correlated with the expression of LIF in both STAD-TGCA and ACRG repositories. Strongly confirming, as shown in our cohort, that a putative regulatory pathway link LIF/LIFR signaling to FGFR4 expression in GC. As mentioned above, this finding might be of therapeutic relevance, since induction of FGFR4 in GC has been essentially linked to the direct transcription of the receptor via protein–protein interaction of *H. pylori* proteins with STAT3. The discovery of an autocrine/paracrine mechanism involved in FGFR4 by the LIF/LIFR signaling is therefore not only novel but of mechanistic relevance. In normal tissues, FGFR4 is activated by binding to the enterokine FGF19 (FGF15 is the mouse ortholog). This pathway is essential for maintaining bile acid and lipid homeostasis in the liver while in cancer cells FGFR4 has been involved in the reprogramming of glucose metabolism in chemoresistant cells, while FGFR4 gene silencing in this setting reverses the enhanced glycolytic flux and chemoresistance [35].

Additionally, since LIF is released both in a paracrine and endocrine manner but is also generated by tumor-infiltrating macrophages (TAM), our results highlight a further mechanism by which oncogenic macrophages regulates cancer cells growth. Indeed, similarly to IL-6, that shares the same half-receptor, GP130, and is released in a TLR4- and NF-KB-dependent manner in response to pro-inflammatory stimuli, including *H. pylori*, LIF is also induced by *H. pylori*-proteins, highlighting the role of ECM-released cytokines in gastric carcinosis and GC growth in the context of *H. pylori* infection [36].

To further elucidate the mechanisms mediating interaction of the LIF/LIFR with FGFR4 we have carried out experiments using in vitro GC cells and hPDOs. The results of these studies demonstrated that exposure of GC cell line, MKN45, to LIF directly promotes FGFR4 expression, both mRNA and protein, and that this effect is mediated by STAT3 activation/phosphorylation.

To mechanistically dissect this pathway, we have synthetized and characterized a novel LIFR inhibitor, LRI-201, designed based on our previous computational studies on mifepristone by manipulating the C11 and C17 position in the estradiene scaffold. Molecular dynamics simulations demonstrated that LRI-201 binds to loops L2 and L3 of the D4 domain of the extracellular region of the hLIFR, altering the structure of the LIF binding site, as shown in Fig. 2R. The analysis of the binding mode of other compounds of the series (Supplementary Figure S5) allowed us to hypothesize that the orientation of the ligand in the binding pocket is driven by the steric hindrance of the aromatic system. Using this newly described agent, we have shown that LIFR antagonism reverses induction FGFR4, mRNA and protein, as well as a decrease in STAT3 phosphorylation caused by exposure of MKN45 cell to LIF, highlighting a mechanistic link between LIF/LIFR and the STAT3-dependent modulation of FGFR4 transcription.

To investigate the mechanistic link between STAT3 and FGFR4 in the context of LIF/LIFR activation we have characterized the STAT3 binding sites on the FGFR4 promoter by a ChIP assay. The results of these experiments demonstrated that STAT3 is recruited to the FGFR4 promoter in response to stimulation of MKN45 cells with LIF, while LIFR inhibition by LRI-201 reversed the binding of phosphorylated STAT3 to promoter of FGFR4. Taken together these results establish a direct link between the LIF/LIFR pathway and the up-regulation of FGFR4 expression in GC cells via phosphorylation and nuclear translocation of STAT3.

The enterokine FGF19 is the physiological ligand of FGFR4. FGF19 is a postprandial hormone that in addition to modulation of glucose and bile acid metabolism, exerts a potent oncogenic effect in liver carcinogenesis [37] and metastasis [38]. Confirming the oncogenic potential of this pathway, exposure of MKN45 cells to FGF19 resulted in a FGFR4-dependent phosphorylation of STAT3 that was reversed by the use of FGFR4 antagonist, roblitinib. Moreover, the inhibition of FGFR4 by roblitinib or its genetic knockdown by transfection of MKN45 with an anti-FGFR4 siRNA partially reversed the effects of LIF on STAT3 phosphorylation and acquisition of an iper-proliferative phenotype, induced by exposure of MKN45 cells to LIF. Additionally, LRI-201 reverted the effect on cell cycle and apoptosis cell rate FGF19 induced. These results highlight the existence of a complex network of regulatory factor that converge on FGFR4 regulation in GC. FGFR4 is overexpressed in the intestinal subtype of GC. There are multiple mechanisms that converge on FGFR4 regulation, and in addition to the direct induction caused by *H. pylori* protein acting on cancer epithelial cells, we have now revealed a regulatory network supported by both FGF19, the natural ligand of FGFR4, and by LIF/LIFR. The present discovery that these two pathways converge in the regulation of STAT3 and its downstream targets in GC cells provides a mechanistic explanation to the positive correlation observed in GC cohorts (Fig. 1) between the expression of LIF and FGFR4 and establishes a regulatory network linking between LIF and FGF19 in FGFR4 regulation in GC. Because the activation of the LIF/STAT3/FGFR4 axis appears to function as a survival mechanism, promoting the growth of tumorigenic cells in response to aberrant expression of LIF and FGFR4 in the intestinal subtype of GC, our study supports the therapeutic potential of anti-LIF based therapies in the treatment of FGFR4 related settings in GC and potentially other neoplastic settings, such as colonic or hepatic tumors.

In conclusion, we have shown that LIF promotes the establishment of an adaptative survival niche in the tumor microenvironment, through the over-phosphorylation of STAT3 leading to FGFR4 overexpression. The LIF/STAT3/FGFR4 axis can serve as survival mechanism to promote the growth of neoplastic cells and development of cancer cells chemoresistance. Present findings ground the basis for exploiting LIFR antagonists in the treatment of FGFR4-related conditions overcoming limits and side effects of pan-FGFR inhibitors in cancer treatment.

### Supplementary Information

Below is the link to the electronic supplementary material.

Supplementary file1 (TIF 948 KB)

Supplementary file2 (TIF 2959 KB)

Supplementary file3 (TIF 5.95 MB)

Supplementary file4 (TIF 625 KB)

Supplementary file5 (TIF 2982 KB)

Supplementary file6 (TIF 3663 KB)

Supplementary file7 (TIF 670 KB)

Supplementary file8 (TIF 3302 KB)

Supplementary file9 (DOCX 78 KB)

Supplementary file10 (DOCX 16 KB)

## Data Availability

Additional materials and methods, including chemical synthesis, molecular docking, in vitro experiments, histopathological analysis, flow cytometry and statistical analysis, are detailed in the Supplementary Material and Methods.The data generated in this study are publicly available The transcriptomic data obtained from the experimental cohort are publicly avaible in Mendeley Data, https://doi.org/10.17632/v6kws68p8k.1. RNA-seq data that support the findings of this study were obtained from The GSE66229 Dataset from ACRG at [https://www.ncbi.nlm.nih.gov/geo/query/acc.cgi?acc=GSE66229], The TCGA-STAD at [https://portal.gdc.cancer.gov/].
