# Supplementary figures and images for: The leukemia inhibitory factor regulates fibroblast growth factor receptor 4 transcription in gastric cancer
Source: Cell Oncol (Dordr). 2023 Nov 9;47(2):695–710. doi: 10.1007/s13402-023-00893-8 (PMC11090936; doi:10.1007/s13402-023-00893-8)

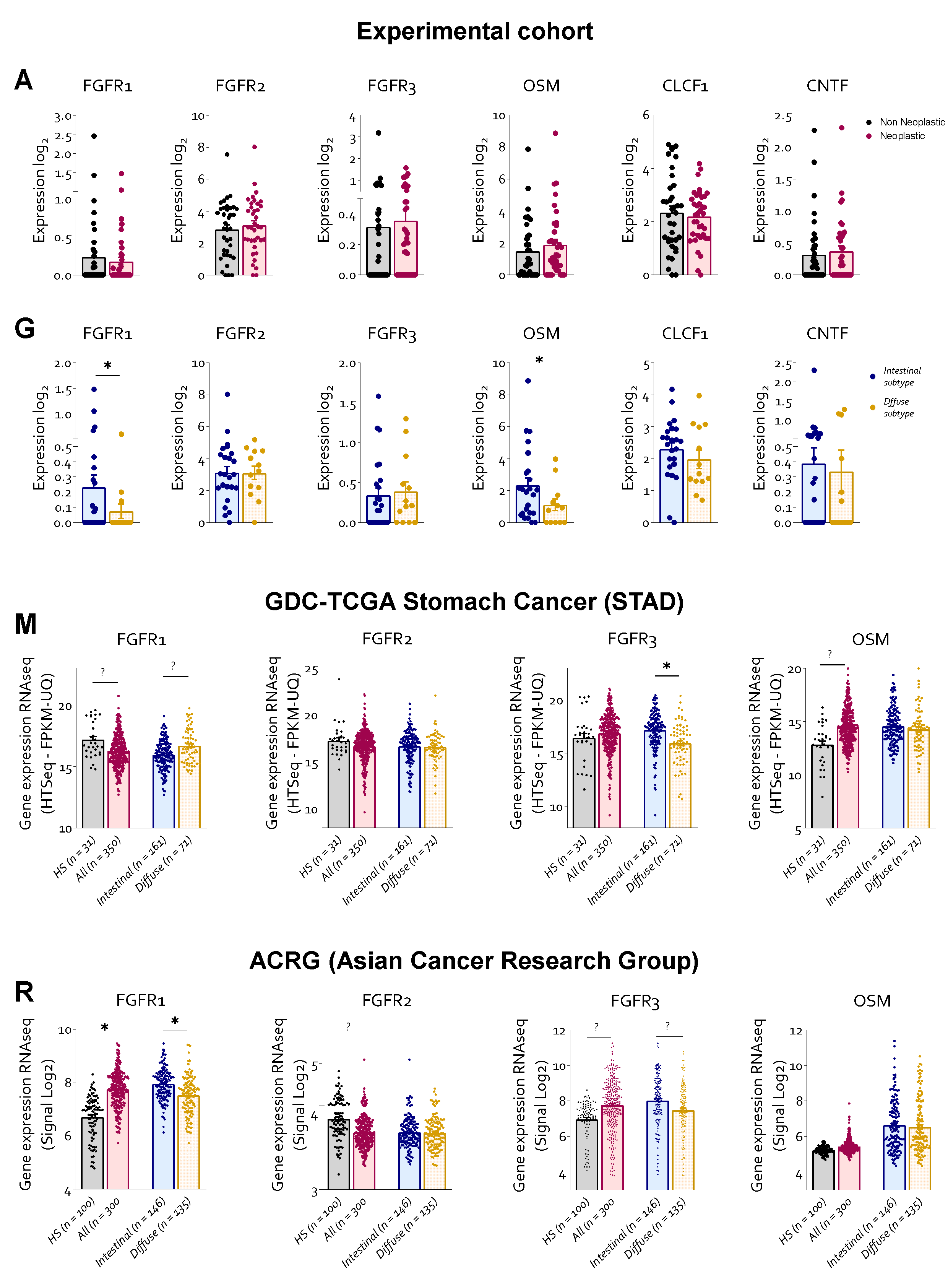

Supplement: Supplementary file 1 — Supplementary file1 (TIF 948 KB) [file 13402_2023_893_MOESM1_ESM.tif]

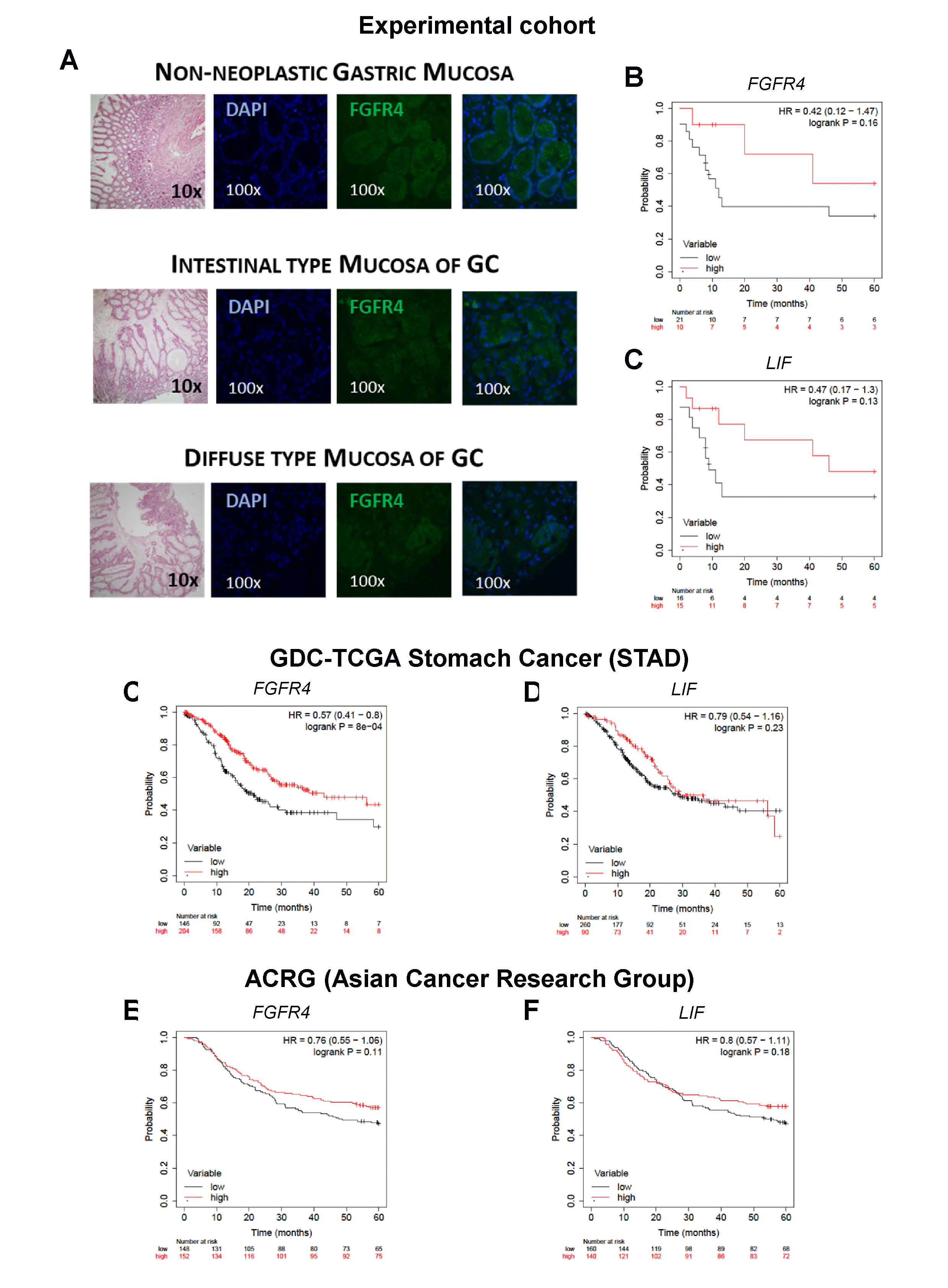

Supplement: Supplementary file 2 — Supplementary file2 (TIF 2959 KB) [file 13402_2023_893_MOESM2_ESM.tif]

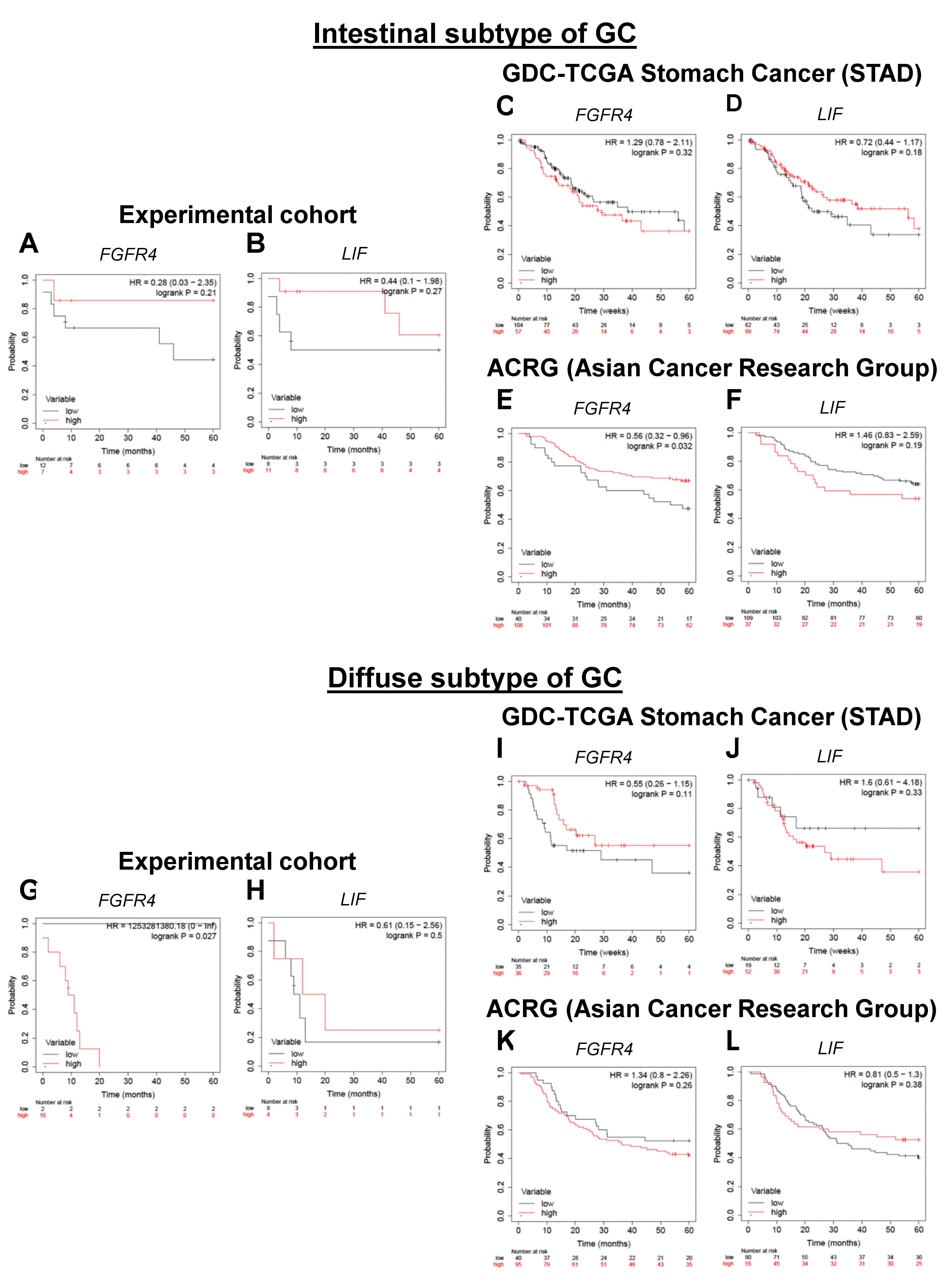

Supplement: Supplementary file 3 — Supplementary file3 (TIF 5.95 MB) [file 13402_2023_893_MOESM3_ESM.tif]

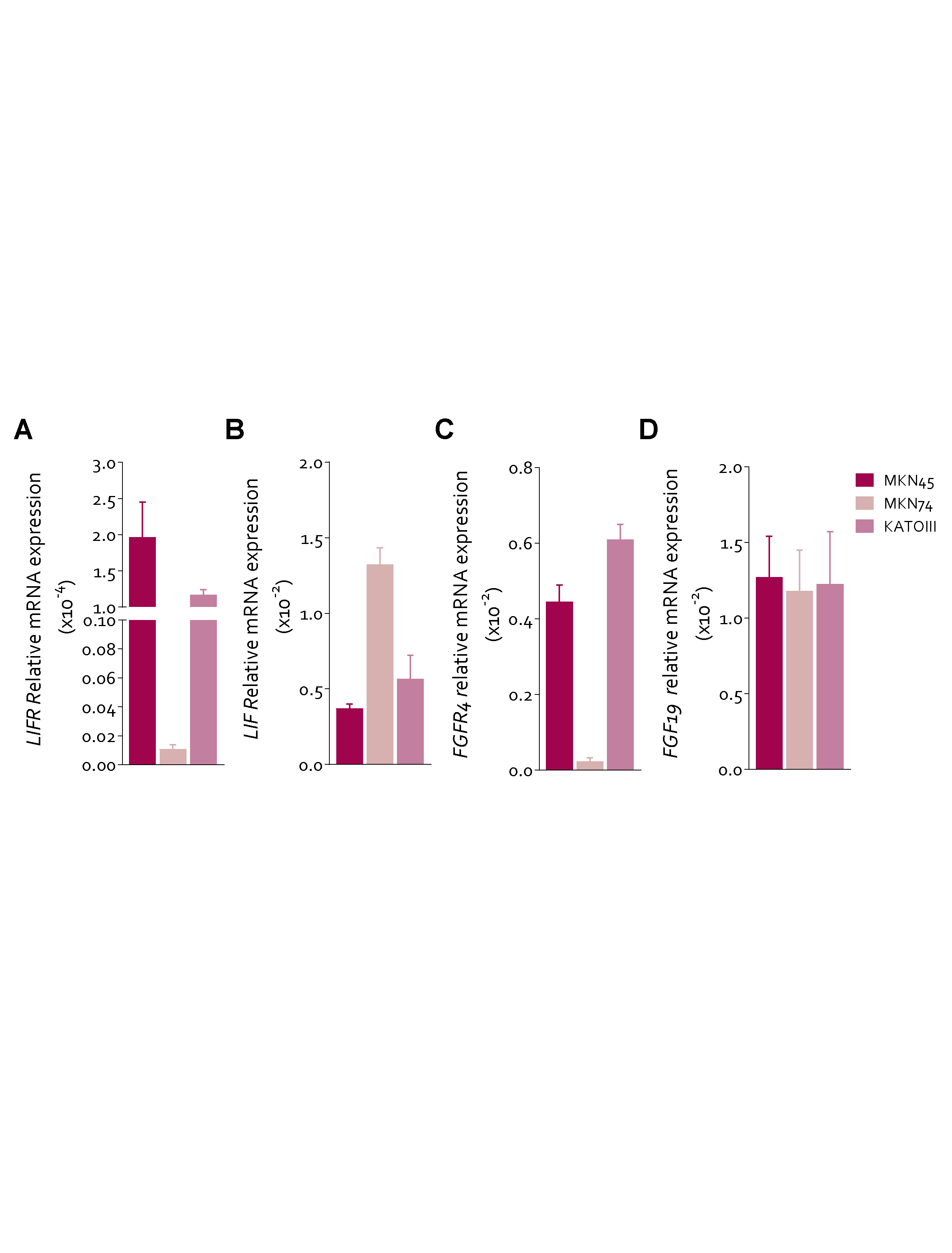

Supplement: Supplementary file 4 — Supplementary file4 (TIF 625 KB) [file 13402_2023_893_MOESM4_ESM.tif]

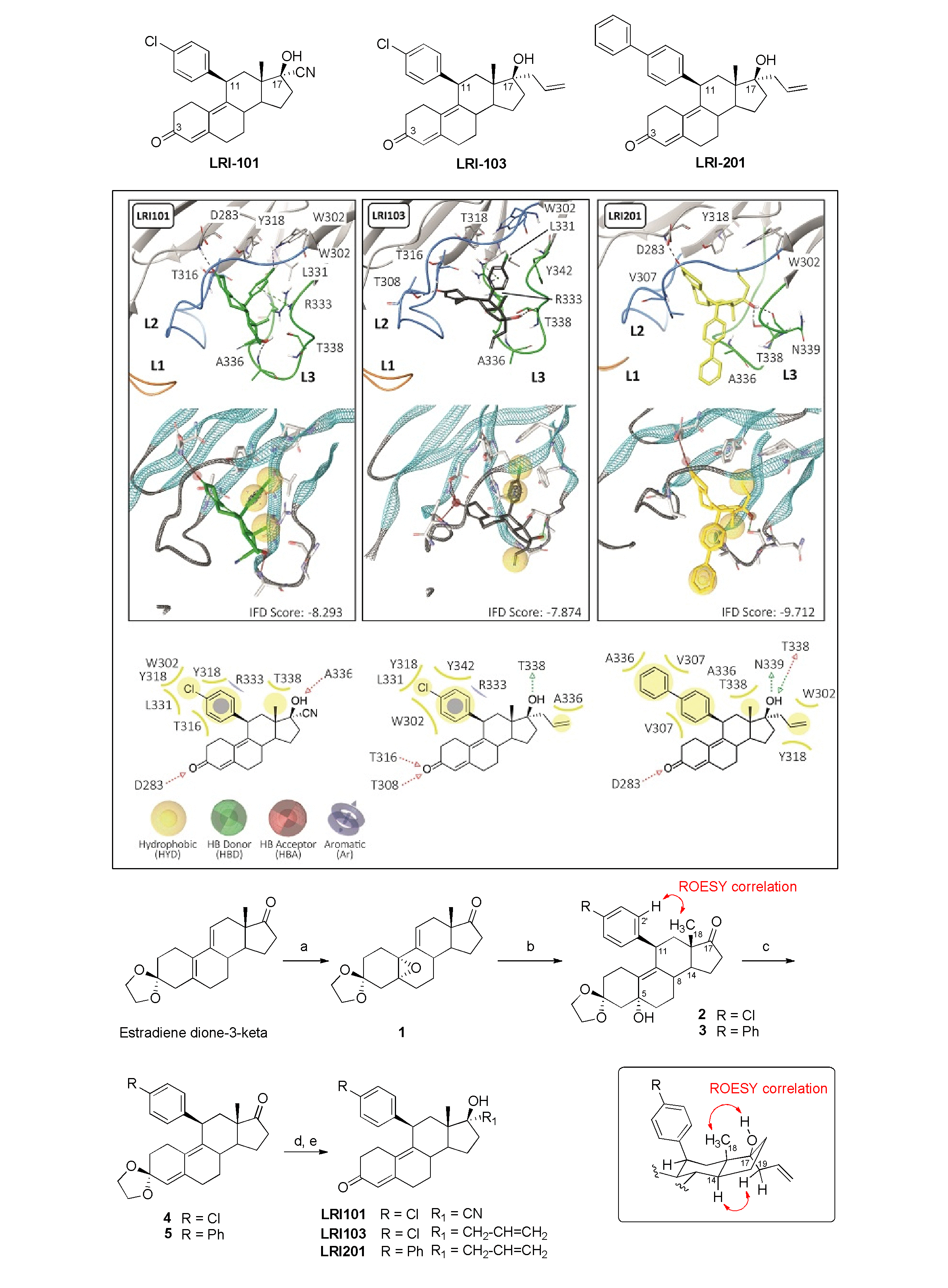

Supplement: Supplementary file 5 — Supplementary file5 (TIF 2982 KB) [file 13402_2023_893_MOESM5_ESM.tif]

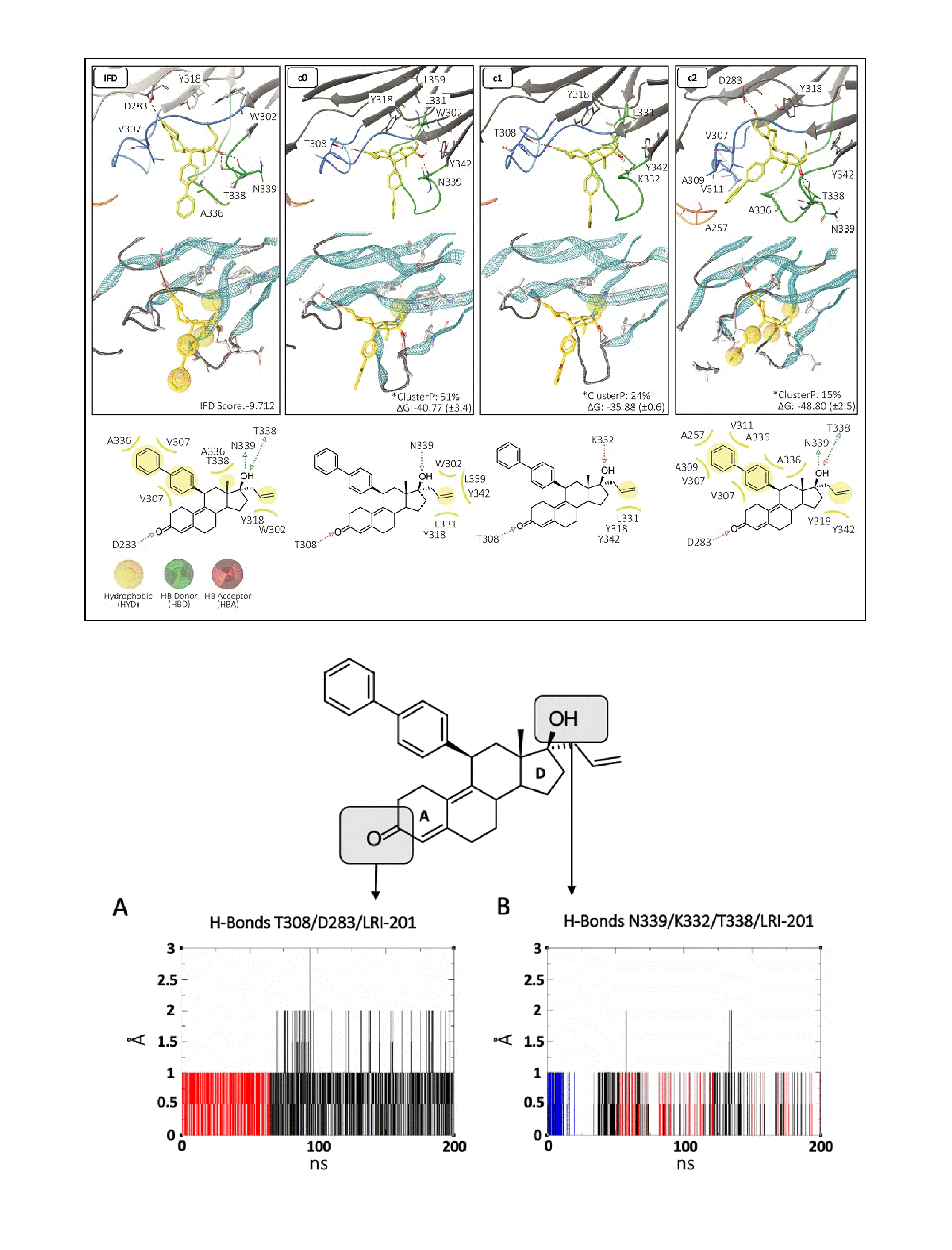

Supplement: Supplementary file 6 — Supplementary file6 (TIF 3663 KB) [file 13402_2023_893_MOESM6_ESM.tif]

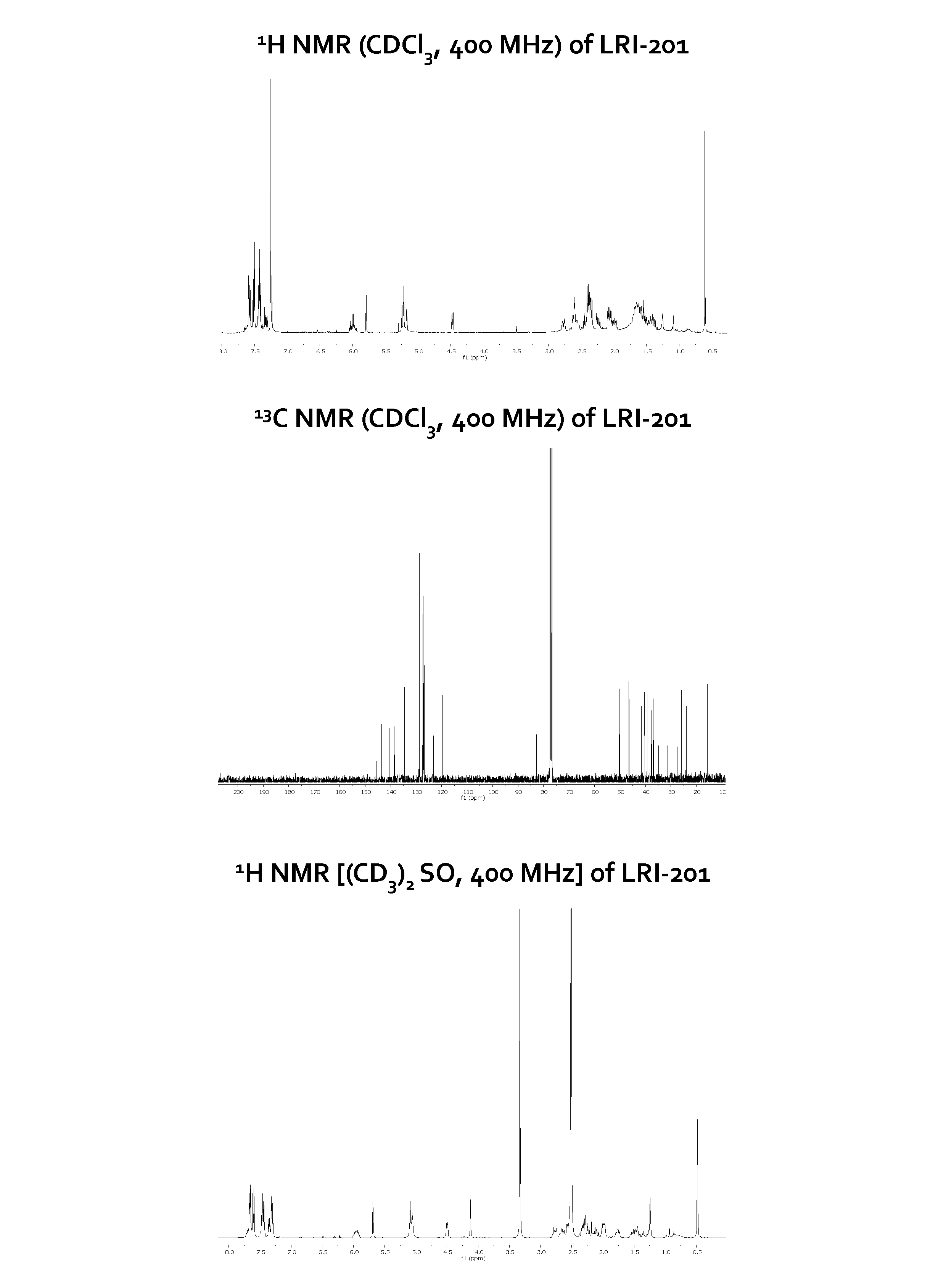

Supplement: Supplementary file 7 — Supplementary file7 (TIF 670 KB) [file 13402_2023_893_MOESM7_ESM.tif]

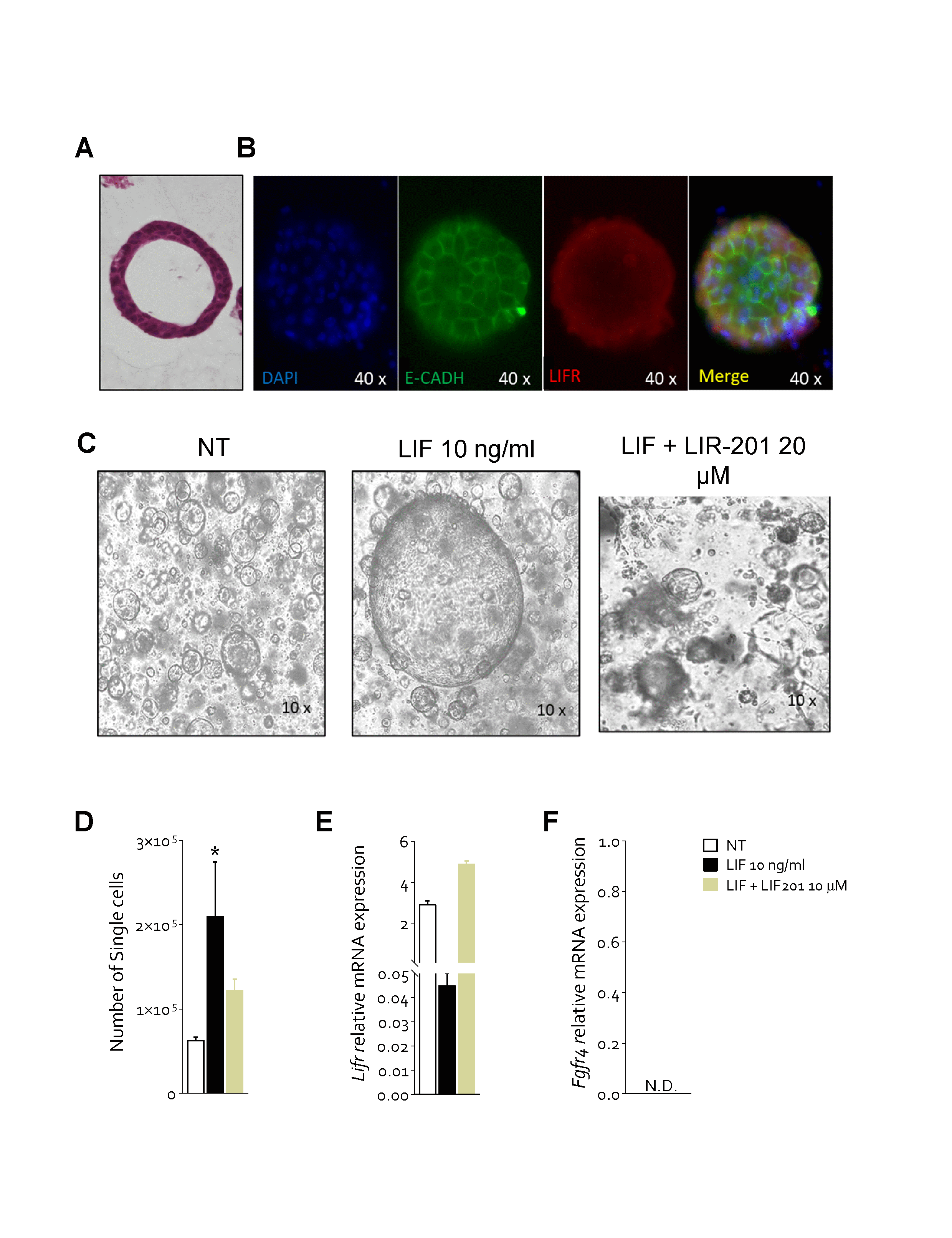

Supplement: Supplementary file 8 — Supplementary file8 (TIF 3302 KB) [file 13402_2023_893_MOESM8_ESM.tif]
