# Supplementary material for: The leukemia inhibitory factor regulates fibroblast growth factor receptor 4 transcription in gastric cancer
Source: Cell Oncol (Dordr). 2023 Nov 9;47(2):695–710. doi: 10.1007/s13402-023-00893-8 (PMC11090936; doi:10.1007/s13402-023-00893-8)
Supplement: Supplementary file 9 — Supplementary file9 (DOCX 78 KB) [file 13402_2023_893_MOESM9_ESM.docx]

**The Leukemia Inhibitory Factor Regulates Fibroblast Growth Factor Receptor 4 Transcription In Gastric Cancer**

Running title

**LIF/FGFR4 pathway in gastric cancer**

Cristina Di Giorgio^1^, Rachele Bellini^1^, Antonio Lupia^2,3^, Carmen Massa^1^, Ginevra Urbani^1^, Martina Bordoni^1^, Silvia Marchianò^1^, Rosalinda Rosselli^4^, Rosa De Gregorio^4^, Pasquale Rapacciuolo^4^, Valentina Sepe^4^, Elva Morretta^5^, Maria Chiara Monti^5^, Federica Moraca^3,4^, Luigi Cari^1^, Khan Rana Sami Ullah^1^, Nicola Natalizi^6^, Luigina Graziosi^6^, Eleonora Distrutti^6^, Michele Biagioli^1^, Bruno Catalanotti^4^, Annibale Donini^1^, Angela Zampella^4^ and Stefano Fiorucci^1^

1 University of Perugia, Department of Medicine and Surgery, Perugia, Italy

2 University of Cagliari, Department of Life and Environmental Sciences, Cagliari, Italy

3 Net4Science srl, University “Magna Græcia", Campus Salvatore Venuta,  Viale Europa, Catanzaro, 88100, Italy

4 University of Naples Federico II, Department of Pharmacy, Naples, Italy

5 University of Salerno, Department of Pharmacy, Salerno, Italy

6 Azienda Ospedaliera di Perugia, Perugia, Italy

**Corresponding Autor: Prof. Stefano Fiorucci**

Stefano Fiorucci

University of Perugia Medical School

Department Surgical and Biomedical Sciences

Tel. +390755858120

Email: stefano.fiorucci @unipg.it

**Supplementary Material and Methods**

**The Kaplan-Meier curve survival**

The Kaplan-Meier Plotter (KM plotter) server [1] was employed to investigate the association between gene expression and survival outcomes. Gene expression data and corresponding survival information from our patient cohort, as well as from TCGA-STAD and GSE66229 series from the ACRG repositories, were uploaded onto the platform and analyzed using the Auto selection protocol to determine the optimal cutoff value [2]; then, Kaplan-Meier plots were generated and downloaded.

**Histological techniques**

*Hematoxylin and Eosin (H&E)*

For histological examination, portions of biopsies of diffuse and intestinal gastric cancers, belonging to patients undergoing surgical resection at the Santa Maria della Misericordia Hospital of Perugia, were fixed in 10% formalin, embedded in paraffin and sectioned. Gastric cancer’s sections were then stained with Hematoxylin/Eosin (H&E), for morphometric analysis.

*Immunofluorescence analysis (IF)*

Immunofluorescence staining was achieved on both biopsies of gastric cancer mentioned above and on MKN45 cell line.

The paraffin-embedded tissue sections were hydrated and then subjected to antigen retrieval by incubation of the slides for 90 min in 95 °C sodium citrate buffer (pH 6.0) and 30 min of cooling at room temperature. After washing 3 times in H_2_O_b_ and 1 time in phosphate buffered saline (PBS 1X), slides have been incubated with Blocking Buffer (PBS 1X, 10% horse serum and 1% BSA) for 1 h at room temperature.

Primary antibodies, anti-LIF-R (ab235908), (Abcam, Cambridge, UK) and anti-FGFR4 (MA5-15500, (Invitrogen, Thermofisher scientific Waltham, Massachusetts, USA), have been prepared in Blocking Buffer and incubated overnight at 4 °C.

The next day, after washing 3 times with PBS 1X containing 0,1 % Tween 20 (PBST), slides were incubated with secondary antibody Goat anti-rabbit IgG (H + L) Alexa Fluor 568 (A11011) for LIF-R and with Goat anti-mouse Ig G (H + L) Alexa Fluor plus 488 (A32723) (Invitrogen, Thermofisher scientific Waltham, Massachusetts, USA) for FGFR4, diluted in Blocking Buffer for 1 h at room temperature in the dark.

# After washing 3 times with PBST, samples have been incubated with Sudan Black for 5 min to quench autofluorescence and then nucleus were counterstained with DAPI 1X for 1 min in the dark and the reaction was stopped with a final wash in PBS 1X for 5 min.

# Slides were mounted with ProLong Glass Antifade Mountant (P36980) (Invitrogen, Thermofisher scientific Waltham, Massachusetts, USA), sealed with nail polish and observed at fluorescence microscope Olympus BX60.

MKN45 cells alone or stimulated with LIF (10 ng/mL), and in another experimental settings also with FGF19 (25 ng/mL) and FGF19 + Roblitinib (20 µM) were plated on slides using Cytospin. Sposts obtained were fixed in methanol for 20 minutes and then submitted at the same procedure of immunofluorescence previously described.

Immunofluorescence was also performed on organoids obtained from neoplastic mucosa of GC patients.

In brief, medium was removed, organoids were washed rapidly once with PBS 1X and fixed in 4% PFA for 20 minutes at RT. After fixation, 4% PFA was removed and organoids were washed, gently rocking, with PBS for 3 times, 5 minutes each.

Organoids were then permeabilized with PBS + 0,5% Triton X-100 for 15 minutes at RT, then washed, gently rocking with IF buffer (PBS+ 0,2% Triton X-100 + 0,05% Tween-20) for 3 times, 5 minutes each, and finally blocked for 1 h with IF buffer + 2,5% BSA at RT.

The primary antibodies (LIFR Abcam ab235908, 1:100; FGFR4 Invitrogen MA5-15500, 1:100) were diluted in IF buffer + 1% BSA and incubated overnight at 4° C.

The next day, primary antibodies were recovered and organoids were washed, gently rocking, in IF buffer for 3 times, 5 minutes each. Organoids were then incubated at RT for 2 h with secondary antibodies Goat Anti-Rabbit IgG H&L Alexa Fluor® 488 (Abcam ab150077) and Goat Anti-Mouse IgG (H+L) Alexa Fluor® plus 488 (Invitrogen A32723) diluted in IF buffer + 1% BSA. After washing 3 times, gently rocking, 5 minutes each, with IF buffer, nuclei were counterstained with DAPI, incubated for 5 minutes at RT. After the last wash, gently rocking, for 5 minutes in IF buffer, organoids are ready for acquisition at the Nikon Eclipse Ti Confocal Spinning Disc CrestV2 or can be stored at 4°C in PBS+NaN.

*Cell fluorescence quantification*

The LIFR and FGFR4 signals in the immunofluorescence images on MKN45 cells were then quantified using the "Measuring cell fluorescence using ImageJ" protocol (<https://sciencetechblog.files.wordpress.com/2011/05/measuring-cell-fluorescence-using-imagej.pdf>) which allows to calculate the corrected total cell fluorescence (CTCF).

**RNA extraction**

RNA was extracted from sample of gastric cancer’s mucosa (intestinal and diffuse type) and from healthy mucosa using TRI reagent (Zymo Research) and Direct-zol™ RNA MiniPrep w/ Zymo-Spin™ IIC Columns (Zymo Research, Irvine, CA) and then used for RNAseq analysis.

RNA from human GC cell lines was extracted using The kit Direct-zol™ RNA MiniPrep w/ Zymo-Spin™ IIC Columns (Zymo Research, Irvine, CA), similarly RNA from human GC cell lines of and from human patient derived oranoids (hPDOs), was obtained using the Direct-zol™ RNA MicroPrep w/ Zymo-Spin™ IIC Columns (Zymo Research, Irvine, CA). RNA extracted was used for qPCR analysis.

**Reverse transcription of mRNA** **and** **Real time (RT)-PCR**

After purification from genomic DNA using DNase I (Thermo Fisher Scientific, Waltham, MA), 1 μg of RNA was reverse transcribed using random hexamer primers with SuperScript II (Thermo Fisher Scientific, Waltham, MA) in a 20-μL reaction volume; 10 ng of cDNA was amplified in a 20-μL solution containing 200 nM each primer and 10 μL of SYBR Select Master Mix (Thermo Fisher Scientific, Waltham, MA). All reactions were performed in triplicate using the following thermal cycling conditions: 3 min at 95 °C, followed by 40 cycles of 95 °C for 15 s, 56 °C for 20 s, and 72 °C for 30 s, using a StepOnePlus system (Applied Biosystems, Foster City, CA). The relative mRNA expression was calculated accordingly to the ΔCt method. Primers were designed using the software PRIMER3 (http://frodo.wi.mit.edu/primer3/) using published data obtained from the NCBI database. The primers used for mouse genes were as following (forward and reverse):

hLIFR (for GCTCGTAAAATTAGTGACCCACA; rev GCACATTCCAAGGGCATATC);

hLIF (for CCCTGTCGCTCTCTAAGCAC; rev GGGATGGACAGATGGACAAC);

hFGFR4 (for GGCCAGGTAGTACGTGCAGA; rev GGCCAGGTCCTTGTCAGAG);

hFGF19 (for CGTGCGGTACCTCTGCAT; rev ATCTCCTCCTCGAAAGCACA);

hKI67 (for TGCTCTGGGTTACCTGGTCT; rev CAAAGGACACACGCCTTCTT);

hBCL2 (for GAAACTTGACAGAGGATCATGC; rev TCTTTATTTCATGAGGCACGTT);

hVIM (for TCAGAGAGAGGAAGCCGAAA; rev ATTCCACTTTGCGTTCAAGG);

hSNAIL1 (for ACCCACACTGGCGAGAAG; rev TGACATCTGAGTGGGTCTGG);

hMUC2 (for AGTTCTCCCGCCAGTGCT; rev CTCCAGGTACACCAGGTTCC);

hCMYC (for TTTCGGGTAGTGGAAAACCA; rev CACCGAGTCGTAGTCGAGGT).

**Chemical synthesis**

**Experimental section – General Experimental Information**

All chemicals were obtained from Sigma-Aldrich and solvents and reagents were used as supplied from commercial sources with some exception. Tetrahydrofuran and dichloromethane were distilled from calcium hydride immediately prior to use. All reaction were carried using flame-dried glassware. Reaction progress was monitored via thin-layer chromatography (TLC) on Alugram silica gel G/UV254 plates. The purification of synthetized compounds was carried out by flash chromatography on Biotage® Selekt. NMR spectra were obtained on Bruker 400 spectrometer and recorded in CDCl_3_ (δ_H_= 7.26, δ_C_=77.0 ppm). J are reported in hertz (Hz) and chemical shifts (δ) are in ppm and referred to CHCl_3_ as internal standards. Spin multiplicities are given as s (singlet), d (doublet), t (triplet), or m (multiplet). High-resolution ESI-MS spectra were performed with LTQ-XL equipped with an Ultimate 3000 HPLC system (Thermo Fisher Scientific) mass spectrometer.

*Synthesis of 3,3-Ethylenedioxy-5α,10α-epoxyestr-9,11-en-17-one* (**1**)

Hydrogen peroxide 30 % (2.05 eq) was added in a solution of hexafluoroacetone trihydrate (1.2 eq) in *dry* CH_2_Cl_2_ at 0 °C. Solid Na_2_HPO_4_ (1.2 eq) was added, and the mixture stirred at 0 °C for 20 min. Then a solution of estradiene dione-3-keta in *dry* CH_2_Cl_2_ was added dropwise over a period of 20 min. The reaction mixture was stirred at 0-4°C for 15 h. Then the reaction was extracted with CH_2_Cl_2_ (3x). The organic fractions were washed with Na_2_SO_3_, saturated NaHCO_3_ solution, and brine. The organic layers were dried over anhydrous Na_2_SO_4_, filtered, and evaporated *in vacuo*. The pure 5α,10α-epoxide **1** was isolated through re-crystallization of the residue from diethyl ether (72% yield). Selected ^1^H NMR (CDCl_3_, 400 MHz): *δ* 6.06 (1H, s, H-11), 3.97-3.88 (4H, m, -OCH_2_CH_2_O-), 0.88 (3H, s, CH_3_-18). HR ESIMS *m/z* 331.1907 [M+H] ^+^, C_20_H_27_O_4_ requires 331.1904.

**Grignard Reaction**

In a solution of **1** in *dry* THF was added CuCl (0.9 eq). The reaction mixture was flushed with N_2_, and then cooled to 0 °C. (4-chlorophenyl)magnesium bromide or [1,1'-biphenyl]-4-ylmagnesium bromide solution (0.5 M, 3 eq) was added dropwise over a period of 15 min. The reaction mixture was stirred at RT for 4 h. Then, the reaction was cooled and quenched by the addition of a saturated NH_4_Cl solution. The reaction was extracted with ethyl acetate (x3) and the organic layers were washed with brine, dried over Na_2_SO_4_, filtered, and concentrated *in vacuo*.

*11β-(4-chlorophenyl)-3,3-ethylenedioxy-5*α*-hydroxyestra-9-en-17-one* (**2**)

The pure adduct was obtained through flash chromatography (hexanes/ethyl acetate 6:4). Selected ^1^H NMR (CDCl_3_, 400 MHz): δ 7.22 (2H, d, *J* = 8.6 Hz, H3’ and H5’), 7.15 (2H, d, *J* = 8.6 Hz, H2’ and H6’), 4.28 (1H, d, *J* = 7.3 Hz, H-11), 4.05-3.89 (4H, m, -OCH_2_CH_2_O-), 0.49 (3H, s, CH_3_-18). HR ESIMS *m/z* 443.1989 [M+H] ^+^, C_26_H_32_ClO_4_ requires 443.1984.

*11β-Biphenyl-3,3-ethylenedioxy-5*α*-hydroxyestra-9-en-17-one* (**3**)

The pure adduct was obtained through flash chromatography (hexanes/ethyl acetate 6:4). Selected ^1^H NMR (CDCl_3_, 400 MHz): δ 7.58 (2H, d, *J* = 9.0 Hz, H2’’ and H6’’), 7.51 (2H, d, *J* = 8.0 Hz, H3’ and H5’), 7.43 (2H, d, *J* = 9.0 Hz, H3’’ and H5’’), 7.32 (1H, t, *J* = 7.6 Hz, H4’’), 7.25 (2H, d, *J* = 8.0 Hz, H2’ and H6’), 4.47 (1H, d, *J* = 7.1 Hz, H-11), 3.97-3.88 (4H, m, -OCH_2_CH_2_O-), 0.60 (3H, s, CH_3_-18). HR ESIMS *m/z* 485.2690 [M+H] ^+^, C_32_H_37_O_4_ requires 485.2686.

*Synthesis of 11β-(4-chlorophenyl)-3,3-ethylenedioxyestra-4,9-dien-17-one* (**4**) and *11β-Biphenyl-3,3-ethylenedioxyestra-4,9-dien-17-one* (**5**)

Acetic anhydride (5 eq) and DMAP (0.1 eq) were added to a solution of **2** or **3** in pyridine, alternatively. The mixture was heated at 65 °C for 24 h.

*11β-(4-chlorophenyl)-3,3-ethylenedioxyestra-4,9-dien-17-one* (**4**). The pure product was purified by flash chromatography using basic alumina in a mixture of hexanes/ethyl acetate (6:4). (51% in two steps) ^1^H NMR selected (CDCl_3_, 400 MHz) δ 7.23 (2H, d, *J* = 8.2 Hz, H3’ and H5’), 7.10 (2H, d, *J* = 8.2 Hz, H2’ and H6’), 5.78 (1H, s, H-4), 4.37 (1H, d, *J* = 7.2 Hz, H-11), 3.97-3.80 (4H, m, -OCH_2_CH_2_O-), 0.52 (3H, s, CH_3_-18). HR ESIMS *m/z* 425.1875 [M+H] ^+^, C_26_H_30_ClO_3_ requires 425.1878.

*11β-Biphenyl-3,3-ethylenedioxyestra-4,9-dien-17-one* (**5**). The pure product was purified by flash chromatography using basic alumina in a mixture of hexanes/ethyl acetate (6:4). (45% in two steps) ^1^H NMR selected (CDCl_3_, 400 MHz) δ 7.58 (2H, d, *J* = 9.0 Hz, H2’’ and H6’’), 7.51 (2H, d, *J* = 8.0 Hz, H3’ and H5’), 7.43 (2H, t, *J* = 9.0 Hz, H3’’ and H5’’), 7.32 (1H, t, *J* = 7.6 Hz, H4’’), 7.25 (2H, d, *J* = 8.0 Hz, H2’ and H6’), 5.82 (1H, s, H-4), 4.47 (1H, d, *J* = 7.1 Hz, H-11), 3.97-3.88 (4H, m, -OCH_2_CH_2_O-), 0.60 (3H, s, CH_3_-18). HR ESIMS *m/z* 467.2586 [M+H] ^+^, C_32_H_35_O_3_ requires 467.2581.

*Synthesis of 17α-cyano-11β-(4-chlorophenyl)-17β-hydroxyestra-4,9-dien-3-one* (**LRI-101**)*, 17α-Allyl-11β-(4-chlorophenyl)-17β-hydroxyestra-4,9-dien-3-one* (**LRI-103**)*, and 17α-Allyl-11β-biphenyl-17β-hydroxyestra-4,9-dien-3-one* (**LRI-201**)

The pure product (**5** or **6**, alternatively) was dissolved in *dry* THF and the reaction mixture was flushed with N_2_ (2x). Then, the reaction was cooled to 0 °C and nucleophilic solution (acetone cyanohydrin or allylmagnesium bromide, alternatively, 3 eq) was added dropwise over a period of 10 min. Then, the reaction was cooled and quenched by the addition of sat. NH_4_Cl solution. The reaction was extracted with ethyl acetate (3x), dried over Na_2_SO_4_, filtered, and concentrated *in vacuo*. The product was used for the next reaction without purification. The compound was dissolved in 10 mL of MeOH/THF 1:1. A 6N solution of HCl (6 eq) was added dropwise and stirred for 1h. The reaction was quenched by adding a sat. NaHCO_3_ solution and extracted with ethyl acetate (3x). The organic layers were washed with water and brine, dried over Na_2_SO_4_, filtered, and concentrated *in vacuo*.

*17α-cyano-11β-(4-chlorophenyl)-17β-hydroxyestra-4,9-dien-3-one* (**LRI-101**). The crude was purified by flash chromatography in hexanes/acetate 7:3 to get a white powder of **LRI-101** (15%). Selected ^1^H NMR (CDCl_3_, 400 MHz): δ 7.20 (2H, d, *J* = 8.8 Hz, H-2’ and H6’), 7.0 (2H, d, *J* = 8.8 Hz, H-3’ and H-5’), 5.79 (1H, s, H-4), 4.46 (1H, d, *J* = 6.7 Hz, H-11), 0.61 (3H, s, CH_3_-18). HR ESIMS *m/z* 408.1721 [M+H] ^+^, C_25_H_27_ClNO_2_ requires 408.1725.

*17α-Allyl-11β-(4-chlorophenyl)-17β-hydroxyestra-4,9-dien-3-one* (**LRI-103**). The crude was purified by flash chromatography in hexanes/acetate 7:3 to get a white powder of **LRI-103** (17%). Selected ^1^H NMR (CDCl_3_, 400 MHz): δ 7.24 (2H, d, *J* = 8.7 Hz, H-2’ and H6’), 7.11 (2H, d, *J* = 8.7 Hz, H-3’ and H-5’), 5.97 ( 1H, m, H-20), 5.78 (1H, s, H-4), 5.22 (1H, dd, *J* = 10.1, 2.0 Hz, H-21a), 5.18 (1H, dd, *J* = 17.2, 2.0 Hz, H-21b), 4.38 (1H, d, *J* = 7.1 Hz, H-11), 0.55 (3H, s, CH_3_-18). HR ESIMS *m/z* 423.2083 [M+H] ^+^, C_27_H_32_ClO_2_ requires 423.2085.

*17α-Allyl-11β-biphenyl-17β-hydroxyestra-4,9-dien-3-one* (**LRI-201**). The crude was purified by flash chromatography in hexanes/acetate 1:1 to get a white solid of **LRI-201** (36%). ^1^H NMR (CDCl_3_, 400 MHz) δ 7.58 (2H, d, *J* = 9.3 Hz, H-2’’ and H6’’), 7.51 (2H, d, *J* = 8.5 Hz, H-3’ and H-5’), 7.43 (2H, t, *J* = 9.3 Hz, H-3’’ and H5’’), 7.32 (1H, t, *J* = 7.3 Hz, H-4’’), 7.25 (2H, d, *J* = 8.5 Hz, H-2’ and H-6’), 6.00 (1H, m, H-20), 5.22 (2H, m, H-21), 5.82 (1H, s, H-4), 4.47 (1H, d, *J* = 7.1 Hz, H-11), 2.8 (1H, m, H-1), 2.64 (2H, m, H-6), 2.60 (1H, m, H-8), 2.59 (1H, m, H-12), 2.43 (2H, m, H_2_-2), 2.41 (1H, m, Hβ-19), 2.39 (1H, m, H-1), 2.27 (1H, m, Hα-19), 2.09 (2H, m, H-7 and H-12), 2.02 (1H, m, H-16), 1.69 (1H, m, H-15), 1.60 (1H, m, H-16), 1.56 (1H, m, H-14), 1.48 (1H, m, H-7), 1.41 (1H, m, H-15), 0.60 (3H, s, CH_3_-18).

Selected ^1^H NMR [(CD_3_)_2_ SO, 400 MHz] δ 0.48 (3H, s, H-18)), 4.12 (1H, s, OH), 4.49 (1H, d, J= 7.0 Hz, H-11), 5.07 (2H, m, H-21), 5.69 (1H, s, H-4), 5.95 (1H, m, H-20), 7.30 (2H, d, J = 8.0 Hz, H-23 and H-27 ), 7.35 (1H, m, H-31), 7.45 (2H, t, J= 7.7 Hz, H-30 and H-32), 7.61 (2H, d, J = 8.2, H-24 and H-26), 7.66 (2H, d, J= 8 Hz, H-29 and H33).

^13^C NMR (CDCl_3_, 400 MHz) δ 15.86 (C-18), 24.11 (C-15), 25.90 (C-1), 27.75 (C-7), 31.26 (C-6), 34.70 (C-16), 37.01 (C-2), 37.50 (C-12), 39.49 (C-8), 40.60 (C-11), 41.75 (C19), 46.58 (C-13), 50.33 (C-14), 82.75 (C-17), 119.64 (C-21), 123.23 (C-4), 127.03 (C-29 and C-33), 127.19 (C-23 and C-27), 127.29 (C24 and C-26), 127.45 (C-31), 128.93 (C-30 and C-32), 129.84 (C-22), 134.65 (C-20), 138.70 (C-28), 140.75 (C-25), 143.61 (C-9), 145.95 (C-10), 156.81 (C-5), 199.67 (C-3).

HR ESIMS *m/z* 465.2785 [M+H] ^+^, C_33_H_37_O_2_ requires 465.2788.

**Computational Studies**

**Ligand and protein preparations.** Both ligand LRI-201 and the x-ray crystal structures of the human LIFR, hLIFR (Uniprot ID Code: P42702, PDB code 3E0G [3], retrieved from the RCSB Protein Data Bank ([www.rcsb.org](http://www.rcsb.org)), were optimized with the same protocol as previously described [4]. The images were rendered using Maestro GUI Suite 2021-1 (Schrödinger Release 2021-1) , LigandScout 4.9 [5] to visualize the key pharmacophoric features in all complexes, and Adobe Illustrator (Adobe Systems, San Jose, CA, USA).

**Docking procedures.**

Two steps of docking studies were carried out on the predicted binding pocket of hLIFr, following the same procedures as described in our previous works [4,6]. In particular, the accurate QM-Polarized Ligands Docking (QPLD) [7] and Induced Fit Docking (IFD) [8] were adopted. After visual inspection of the QPLD result, the best pose was refined with IFD procedure using the extended sampling protocol. A maximum of 80 poses was generated, and the energy window for the ligand conformational sampling was 2.5 kcal/mol.

**Molecular Dynamics simulation (MDs)**

To investigate the LRI-201 binding stability of the best-scored IFD docking pose, 200 ns of MDs was run using the CUDA version of AMBER18 package [9], using the Amber ff14SB force field [10,11] to treat the protein. The LRI-201 charges were calculated using the restrained electrostatic potential (RESP) fitting procedure [12] Specifically, the Gaussian16 package [13] was used to calculate the ligand ESP using the 6-31G* basis set at the Hartree-Fock level of theory while Antechamber module [14] coupled with the general amber force field (GAFF2) parameters [15] allowed RESP charges and the ligand force field parameters. The SHAKE algorithm was used to constrain bonds involving hydrogen atoms with an integration timestep of 2 fs. The system was solvated in a 10 Å layer of the octahedral box using TIP3P [16] water molecules parameters. Successively, the system was minimized and thermally equilibrated as described in our latest work [6]. A total of 200 ns of MDs production were performed, and the resulting trajectory was visualized by using Visual Molecular Dynamics (VMD) graphics ver. 1.9.3 [17]. Clustering and analysis procedures were carried out through the CPPTRAJ module [18], and the Molecular Mechanics/Generalized Born Surface Area (MM/GBSA) equation [19] was applied for the most representative cluster population.

**Alpha screen assay**

Recombinant human LIFR (His-Tag) and LIF (biotinylated) were purchased from Sino Biologicals (Sino Biological Europe GmbH, Dusseldorf, Germany) and R&D Systems (Abingdon, UK), respectively, and both were reconstituted as required by the manufacturer. Inhibition of LIFR/LIF binding by LRI-101, LRI-103 and LRI-201 was measured by Alpha Screen (Amplified Luminescent Proximity Homogeneous Assay), in white, low-volume, 384-well AlphaPlates (PerkinElmer, Waltham, MA, USA) using a final volume of 25 μL and an assay buffer containing 25 mM Hepes (pH 7.4), 100 mM NaCl, and 0.005% Kathon. The concentration of DMSO in each well was maintained at 5% vol/vol. LIFR (His-Tag, final concentration 4.5 nM) was incubated with three compounds or DMSO for 45 min under continuous shaking. Then, LIF was added (biotinylated, final concentration 9 nM), and the samples were incubated for 15 min prior to adding nickel chelate acceptor beads (final concentration 20 ng/μL) for 30 min. Then, streptavidin donor beads were added (final concentration 20 ng/μL), and the plate was incubated in the dark for 2 h and then read in an EnSpire Alpha multimode plate reader (PerkinElmer, Waltham, MA, USA).

**Transactivation assay**

To perform STAT3 transactivation, HepG2 (HB, 8065 from ATCC), an immortalized human hepatocarcinoma cell line was used, as described previously. On day 0, HepG2 were seeded at 7.5 × 10^4^ cells/well in a 24-well plate and maintained at 37 °C and 5% CO2 in E-MEM supplemented with 10% FBS, 1% glutamine, and 1% penicillin/streptomycin. On day 1, cells were transiently transfected with the reporter plasmid pGL4.47[luc2P/SIE/Hygro] (200 ng) (CAT#: E4041 Promega, Madison, WI, USA), a vector encoding the hLIFR (CAT# RC226327) (100 ng) and CD130 (IL6ST) (100 ng) (CAT#: RC215123, OriGene Technologies, Inc. Rockville, MD, USA), and finally a vector encoding the human RENILLA luciferase gene (pGL4.70) (100 ng) (Promega, Madison, WI, USA). On day 2, cells were exposed to the cytokine LIF (10 ng/mL) alone or in combination with LRI-201 (from 0.1 to 20 μM). Then, after 24 h, the cells were lysed in 100 μL of lysis buffer (25 mM Tris-phosphate, pH 7.8; 2 mM dithiothreitol (DTT); 10% glycerol; 1% Triton X-100). 10 μL cellular lysates were assayed for luciferase and RENILLA activities using the Dual-Luciferase Reporter assay system (Promega, Madison, WI, USA). Luminescence was measured using a Glomax 20/20 luminometer (Promega, Madison, WI, USA). LUCIFERASE activities (RLU) were normalized with RENILLA activities (RRU).

**Protein extraction and Western blotting**

MKN45 were lysed in RIPA lysis buffer containing phosphatase and protease inhibitors cocktail; aliquots from each sample containing 50 µg of protein were separated on Novex WedgeWell 4-12% Tris-Glycine gel (Invitrogen) and transferred to nitrocellulose membrane with iBlot 2 Dry Blotting System (Invitrogen).The blots were subsequently blocked for 1 h with 5% milk powder in Tris-buffered saline (TBS)/Tween 20 at RT and then probed overnight (at 4 °C) with primary antibodies against GAPDH (Cell Signaling D4C6R, 1:1000), LIFR (Abcam ab235908 1:1000 ), FGFR4 (Invitrogen MA5-15500; 1:1000), pFGFR4 (Tyr642) (AF7262, 1:1000 Affinity Biosciences), STAT3 (sc-8019 1:1000; Santa Cruz Biotechnology), pSTAT3 (Genetex GTX118000, 1:1000), JAK1 (D1T6W) (50996, 1:1000, Cell signalling), pJAK1,().After overnight incubation, appropriate horseradish peroxidase-labeled secondary antibody, at a dilution of 1:1000, were used. Positive signals were developed by Immobilon Western Chemiluminescent HRP Substrate (Merck Millipore) and quantitative densitometry analysis was performed using ImageJ Software. The degree of STAT3 phosphorylation was calculated as the ratio between the densitometry readings of GAPDH and p-STAT3/ STAT3.

**Cell proliferation assay**

The cell viability assay was done using the CellTiter 96 Aqueous One Solution Cell Proliferation Assay (Promega, Milano, Italy), a colorimetric method for accessing the number of viable cells in proliferation as described previously. MKN45 cells were seeded in RPMI complete medium at 36 *10^3^ cells/100 uL well into 96-well tissue culture plate. After 24 h, cells were serum starved for 24 h. In the first experimental set cell were primed with the LIFR ligand, LRI-201 at different concentrations (0,1;10; 20; 30; 50 μM). In a second experimental set LIF (10 ng/mL), FGF19 (25 ng/mL), LIF + roblitinib (20 µM) and FGF19 + roblinitinib for 8 h. Then cell proliferation was assessed as mentioned above. Absorbance was measured using a 96 well reader spectrophotometer (490 nm). In these experiments each experimental setting was replicated ten folds. For analysis the background readings with the medium alone, were subtracted from the samples read-outs.

**Flow-cytometry**

MKN45 cells were seeded in 6-well tissue culture plate (cell density 700 × 10^3^/well) and cultured as specified above. Cells were serum-starved for 8 h and then incubated with LIF (10 ng/mL) alone or plus LRI-201 (20 µM) or a vehicle for 24 h. In another experimental set cells were serum-starved for 8 h and then incubated with LIF (10 ng/mL), FGF19 (25 ng/mL), LIF + roblitinib (20 µM) and FGF19 + roblinitinib for 24 h. In the last experimental set, cells were serum-starved for 8 h and then incubated with FGF19 (25 ng/mL) alone or plus LRI-201 (20 µM) for 24 h. The intracellular flow cytometry staining for Ki-67 was performed using the following reagents: Ki-67 Monoclonal Antibody (SolA15), Alexa Fluor™ 488, (eBioscience™, San Diego, California, USA) and 7-AAD to characterize the cell cycle phases G0-G1 and S-G2-M. Before intracellular IC-FACS, staining cells were fixed for 30 min in the dark using IC Fixation buffer (eBioscience™) and then permeabilized using Permeabilization buffer (10X) (eBioscience™). The staining for Annexin V was performed using the Annexin V Antibody (A13199, Thermofisher Scientific, Waltham, MA, USA) to evaluate the apoptosis rate. Briefly, 5 μL of Annexin V and E-cadherin Antibody (Miltenyi™). Antibody was added to each 100 μL of cell suspension, and cells were incubated the at room temperature for 15 min.

Cells were analyzed with FACS Fortessa. Data was analyzed with FlowJo software (Tree Star) and the gates set using a fluorescence minus-one (FMO) control strategy. FMO controls are samples that include all conjugated Abs present in the test samples except for one. The channel in which the conjugated Ab is missing is the one for which the fluorescence minus one provides a gating control.

**Wound healing assay**

MKN45 cells were seeded in RPMI complete medium at 800x10^3^ cells/well into 24-well plate and used at 70-80% confluence rate. The assay was performed as previously described [20], particularly on the day 1, the cell monolayers were gently scraped vertically with a new 0.2 mL pipette tip across the centre of the well. After scratching, the well was gently washed twice with PBS (Euroclone, Milan, Italy) to remove the detached cells and cell debris and finally fresh medium containing LIF (10 ng/mL) alone or in combination with LRI-201 (20 µM) was added into each well. Immediately after scratch creation, the 24-plate was placed under a phase-contrast microscope and the first image of the scratch acquired (T0) with using a OPTIKAM Pro Cool 5 – 4083.CL5 camera. Cells were grown for additional 48 h and images taken at 24h (T1) and 48 h (T2) (data not shown). The gap distance between scarps borders was quantified by assessing that area between the two margins of the scratches. All experiments were performed in triplicate.

**Chromatin immunoprecipitation (ChIP) assay**

For the chromatin immunoprecipitation (ChIP) assay, we analysed the FGFR4 promoter region, which spans from 0 to 5000 bp upstream of exon 1, using the “EPD-The eukaryotic promoter database” and “JASPAR-DNA binding sequence” and we detected four STAT3-inducible elements (SIE) site located respectively: Site 1: from -576 to -587, Site 2: from -828 – 839, Site 3: -4212 -4223, Site 4: -4233 – 4416, upstream of the ATG starting sequence of the gene encoding for hFGFR4. MKN45 cells (2*10^6^) were exposed LIF alone or in combination with LRI-201 (20 μM) for 1 H. ChIP assays were performed according to the manufacturer’s protocols (Pierce Agarose ChIP kit, Thermo fisher). The promoter DNA was quantified by real-time PCR analysis using primers around the SIE in the proximal region of FGFR4 promoter (Site 1 and Site 2), particularly (forward AGCAGAAGGAAGGGGTTCTC, reverse CCCTATACACACCCCATTCAG) and a couple of primers was chosen around the SIE in the distal region of FGFR4 promoter (Site 3 and Site 4), particularly (forward GGGAGCTGAAGAGTCACGAG, reverse ATTACAGGCGTGAGCAACG).

Primers spanning a region located ∼2029 kb downstream of the FGFR4 transcriptional start site (CCGGATTGGAGTGCAGTAAT; rev CTACACACTTGGCAGGCTGA) were used as control. At least three replicates of each group were performed.

**FGFR4 small interfering RNA knockdown**

Stably-transfected cells were obtained by retroviral infection, specifically through pRS vectors containing different shRNAs sequences for FGFR4 gene (TR320356A, TR320356B, TR320356C and TR320356D) plus a scrambled control shRNA (TR30012) purchased from Origene (OriGene Technologies, Inc. USA). Furth more, each plasmid incorporates as well the antibiotic gene resistance, fundamental for the following selection step. Briefly, MKN45 cells were seeded in RPMI complete medium at 3x105 cells per well onto a 6 well tissue culture plate. The day after, they were transiently transfected with 1.2 µg per well of different FGFR4 plasmids or the control vector, using the FuGENE® HD Transfection Reagent from Promega, (1,8 µl/well), into a final transfection volume of 150 µL per well. After an incubation of 24 hours, the same protocol was applied for the second time. The very next day, MKN45 transfected cells were selected using 4 µg/ml of puromycin (Sigma) in fresh RPMI medium. At 72 hours from selection, we amplified cells to repeat the selection 3 times further, until seeding them in RPMI totally free from the antibiotic. The efficacy of the protocol was evaluated through the quantitative reverse-transcription polymerase chain reaction (qRT-PCR), using specific primers for FGFR4: actually, only the TR320356D plasmid resulted effective into gain the silencing.

**Supplementary Results**

In view of the antagonistic activity of the mifepristone on the LIF-LIFR axis, as previously reported [6], we have focused our attention on the simplification and optimization of the synthetic process, manipulating C11 and C17 position in the estradiene scaffold.

Regarding the C17 position, molecular dynamics simulations of mifepristone binding to LIFR showed a clear role of the OH group in establishing H-bond interactions with the hydroxyl group of Thr338 on the loop 3 (L3), while the propynyl group established weak and discontinuous hydrophobic contacts with side chains of Ala336 and Glu335. We therefore replaced the propynyl group with a cyanide group or with an allyl group, to explore the role of electrostatic and hydrophobic interactions with the loop L3. About C11 position, we explored the effects of p-Cl phenyl ring and of a bulkier biphenyl system, which is the substituent on position 11 in EC359, previously identified as LIFR inhibitor [21]. This led to the design of compounds LRI-101, LRI-103 and LRI-201 (Figure S5 panel A). Preliminary two steps docking results yielded for LRI-101 and LRI-103 a binding mode very similar to the one found for mifepristone, with the estradiene scaffold placed in between loops L2 and L3, and the p-Cl-phenyl in position 11 pointing toward the inner core of the D4 domain. Interestingly, the substitution of the single para substituted phenyl ring with a biphenyl resulted in a change of the orientation of the binding mode. Docking results for LRI-201 showed the estradiene scaffold placed between L2 and L3, while the biphenyl system pointed toward the external face of the D4 domain. (Figure S5 panel B).

These results prompted us to synthetize the designed compounds.First, the para-chlorophenyl group at C11 was easily introduced with a Grignard reaction, followed by the installation of the CN group at C17, via classic addition of cyanide to a carbonyl group (LRI-101). Unfortunately, LRI-101 showed very poor thermal and chemical stability over time, resulting a hydrogen cyanide source. Consequently, we thought to replace the alkyne function at C17 with an allyl group, achieving LRI-103 (Figure S5, panel C). Simultaneously, we also increased the hydrophobicity at C11 position, introducing a biphenyl group (LRI-201).

LRI-101-103 were prepared in a five-step procedure, starting from the commercially available estradiene dione-3-keta (Figure S5 panel C). The first step was a chemoselective epoxidation of the Δ5,10 double bond that was achieved with H2O2 and a catalytic amount of hexafluoroacetone to get a mixture of 5,10α-epoxide (**1**) and 5,10β-epoxide in 3:1 ratio with excellent yield. The required 5,10α-epoxide was isolated by recrystallization in diethyl ether with 72% yield.

Opening of the 5,10α-epoxide via SN2 attack to the allylic position at the C11 by Grignard reagents in the presence of a catalytic amount of copper(I) chloride followed by hydroxy group elimination at position 5 with acetic anhydride and DMAP furnished compounds **4** and **5** in 51 and 45% yield over two steps, respectively. The Cu(I)-catalyzed reaction proceeds with excellent regio- and stereoselectivity [22], leading exclusively to the 11β-stereochemistry, that was established by comparison of NMR data with previous reports on a broader range of compounds, on the basis of the diagnostic down-fielded shifted effect in 1H-NMR of methyl group at C18 (δ = 0.60 ppm) and on the careful analysis of ROESY data of compound **5**, showing the dipolar correlation between H-2’ at δ 7.25 ppm and H-18 at δ 0.60 ppm.

Finally, a nucleophilic addition with acetone cyanohydrin or allylmagnesium bromide to carbonyl at C17, and the subsequent acid treatment afforded the LRI-101-103 in a stereoselective manner. Diagnostic ROESY cross-peaks showed in Supplementary Figure S5, panel C and observed in DMSO spectrum confirmed the α-orientation of allyl group at C17 and the structure of compound LRI-201.

**Alphascreen results**

We employed a cell-free system using the Alpha Screen assay, which showed that LRI-101 and LRI-201 effectively inhibited LIF/LIFR interaction with an IC50 of 15.50 ± 2.56 µM and of 21.92 ± 2.16 µM (Table S1), respectively, whereas LRI-103 is the less potent antagonist of the series (IC50 of 38.13 ± 3.56 µM). Since LRI-101 showed very poor thermal and chemical stability, LRI-201 has been selected for further experiments.

**Computational studies on LRI-201**

To disclose the binding mode and the mechanism of action of LRI-201, a two-step docking procedure, followed by 200 ns of Molecular Dynamics simulations (MDs), were performed. Shortly, the initial pose was obtained by docking the LRI-201 structure on the apo structure of the extracellular domains D1-D5 of the human Leukemia Inhibitory Factor Receptor (hLIF-R) (PDB ID 3EOG) with the QM-Polarized Ligand Docking (QPLD) protocol. The best QPLD poses were selected for the Induced Fit Docking (IFD) docking step, including in the ligand binding calculations the flexibility of the hLIF-R loops L1 (255-260), L2 (303-316) and L3 (332-341). Finally, IFD best poses (Supplementary Figures S6, panel A) were further refined by means of 200 ns of classical MDs.

The MDs analysis showed a high flexibility of the binding site, with LRI-201 stable within a pocket formed by loops L2 and L3. The RMSD analysis of the ligand binding (L-RMSD) (Supplementary Figures S6, panel B) showed that LRI-201 assumed a stable binding after about 70 ns.

Furthermore, the cluster analysis revealed that, after 70 ns, LRI-201 binding can be described by two very similar clusters (namely c0 and c1; Table S2 and Supplementary Figures S6, panel B). Despite the L3 high loop flexibility, which causes discontinuous H-bond between the 17-OH group of LRI-201 and the Asn339, Lys332 and Thr338 (Supplementary Figures S6, panel C), the binding mode of the ligand resulting from the main c0 and c1 cluster keeps stable. The essential elements of the binding consisted in a H-bond between the carbonyl group in position 3 and Thr308, that anchored the ring A to loop L2, π-π interactions of the allyl double bond with Tyr318, Leu331 and Tyr342 (that bonded the ring D to the loop L3, and the biphenyl ring pointing toward the LIF binding interface (Figure 1, panel R and Supplementary Figures S6, panel A). Additional hydrophobic interactions of Pro304 and Pro337 with the biphenyl moiety, and of Tyr318 and the methylene chain of Lys332 with the estradiene scaffold, contributed to stabilize the ligand binding of clusters c0 and c1. The only difference between clusters c0 and c1 resided in the H-bonding of the 17-OH group of LRI-201 with Asn339 (c0) or Lys332 (c1) (Supplementary Figures S6, panel C), which is determined by the high flexibility of the loop L3. Therefore, MD simulations depicted a stable binding mode of LRI-201 on the D4 domain, which is characterized by stable interactions with loop L2 residues.

**References**

1. A. Lánczky and B. Győrffy, J. Med. Internet Res. **23**, e27633 (2021).

2. B. Győrffy, GeroScience (2023).

3. G. Skiniotis, P. J. Lupardus, M. Martick, T. Walz, and K. C. Garcia, Mol. Cell **31**, 737 (2008).

4. C. Di Giorgio, R. Bellini, A. Lupia, C. Massa, M. Bordoni, S. Marchianò, R. Rosselli, V. Sepe, P. Rapacciuolo, F. Moraca, E. Morretta, P. Ricci, G. Urbani, M. C. Monti, M. Biagioli, E. Distrutti, B. Catalanotti, A. Zampella, and S. Fiorucci, Front. Oncol. **13**, 1140730 (2023).

5. G. Wolber and T. Langer, J. Chem. Inf. Model. **45**, 160 (2005).

6. C. Di Giorgio, A. Lupia, S. Marchianò, M. Bordoni, R. Bellini, C. Massa, G. Urbani, R. Roselli, F. Moraca, V. Sepe, B. Catalanotti, E. Morretta, M. C. Monti, M. Biagioli, E. Distrutti, A. Zampella, and S. Fiorucci, Cells **11**, (2022).

7. 2021. Schrödinger Release 2021-4: QM-Polarized Ligand Docking protocol; Glide, Schrödinger, LLC, New York, NY, 2021; Jaguar, Schrödinger, LLC, New York, NY, 2021; QSite, Schrödinger, LLC, New York, NY, (n.d.).

8. 2021. Schrödinger Release 2023-1: Induced Fit Docking protocol; Glide, Schrödinger, LLC, New York, NY, 2021; Prime, Schrödinger, LLC, New York, NY, (n.d.).

9. T.-S. Lee, D. S. Cerutti, D. Mermelstein, C. Lin, S. LeGrand, T. J. Giese, A. Roitberg, D. A. Case, R. C. Walker, and D. M. York, J. Chem. Inf. Model. **58**, 2043 (2018).

10. C. I. Bayly, P. Cieplak, W. Cornell, and P. A. Kollman, J. Phys. Chem. **97**, 10269 (1993).

11. J. A. Maier, C. Martinez, K. Kasavajhala, L. Wickstrom, K. E. Hauser, and C. Simmerling, J. Chem. Theory Comput. **11**, 3696 (2015).

12. Y. J. Kim, S. H. Jeong, E.-K. Kim, E. J. Kim, and J. H. Cho, Oncol. Rep. **38**, 3632 (2017).

13. M. J. Frisch, G. W. Trucks, H. B. Schlegel, G. E. Scuseria, M. a. Robb, J. R. Cheeseman, G. Scalmani, V. Barone, G. a. Petersson, H. Nakatsuji, X. Li, M. Caricato, a. V. Marenich, J. Bloino, B. G. Janesko, R. Gomperts, B. Mennucci, H. P. Hratchian, J. V. Ortiz, a. F. Izmaylov, J. L. Sonnenberg, Williams, F. Ding, F. Lipparini, F. Egidi, J. Goings, B. Peng, A. Petrone, T. Henderson, D. Ranasinghe, V. G. Zakrzewski, J. Gao, N. Rega, G. Zheng, W. Liang, M. Hada, M. Ehara, K. Toyota, R. Fukuda, J. Hasegawa, M. Ishida, T. Nakajima, Y. Honda, O. Kitao, H. Nakai, T. Vreven, K. Throssell, J. a. Montgomery Jr., J. E. Peralta, F. Ogliaro, M. J. Bearpark, J. J. Heyd, E. N. Brothers, K. N. Kudin, V. N. Staroverov, T. a. Keith, R. Kobayashi, J. Normand, K. Raghavachari, a. P. Rendell, J. C. Burant, S. S. Iyengar, J. Tomasi, M. Cossi, J. M. Millam, M. Klene, C. Adamo, R. Cammi, J. W. Ochterski, R. L. Martin, K. Morokuma, O. Farkas, J. B. Foresman, and D. J. Fox, Gaussian 16 (2016).

14. J. Wang, W. Wang, P. A. Kollman, and D. A. Case, J. Mol. Graph. Model. **25**, 247 (2006).

15. X. He, V. H. Man, W. Yang, T.-S. Lee, and J. Wang, J. Chem. Phys. **153**, 114502 (2020).

16. W. Jorgensen, J. Chandrasekhar, J. Madura, R. Impey, and M. Klein, J. Chem. Phys. **79**, 926 (1983).

17. W. Humphrey, A. Dalke, and K. Schulten, J. Mol. Graph. **14**, 33 (1996).

18. D. R. Roe and T. E. 3rd Cheatham, J. Chem. Theory Comput. **9**, 3084 (2013).

19. S. Genheden and U. Ryde, Expert Opin. Drug Discov. **10**, 449 (2015).

20. C. Di Giorgio, S. Marchianò, E. Marino, M. Biagioli, R. Roselli, M. Bordoni, R. Bellini, G. Urbani, A. Zampella, E. Distrutti, A. Donini, L. Graziosi, and S. Fiorucci, Front. Oncol. **12**, 939969 (2022).

21. S. Viswanadhapalli, Y. Luo, G. R. Sareddy, B. Santhamma, M. Zhou, M. Li, S. Ma, R. Sonavane, U. P. Pratap, K. A. Altwegg, X. Li, A. Chang, A. Chávez-Riveros, K. V Dileep, K. Y. J. Zhang, X. Pan, R. Murali, M. Bajda, G. V Raj, A. J. Brenner, V. Manthati, M. K. Rao, R. R. Tekmal, H. B. Nair, K. J. Nickisch, and R. K. Vadlamudi, Mol. Cancer Ther. **18**, 1341 (2019).

22. G. Teutsch and A. Bélanger, Tetrahedron Lett. **20**, 2051 (1979).

**Supplementary Figures**

**Figure S1.** Transcriptome analysis of paired non neoplastic and neoplastic tissues in 31 patients with GC. Gene expression (Log2) of: **A)** FGFR1, FGFR2, FGFR3 and LIFR ligands, OSM CLCF1 and CNTF **A)** in non-neoplastic and neoplastic mucosa; **B)** in intestinal vs diffuse histological subtype. RNA-seq analysis of healty gastric mucosa and neoplastic mucosa from GC patient obtained from external databases STAD-TGCA and ACRG. Gene expression of: FGFR1, FGFR2, FGFR3 and LIFR ligands, OSM from **C)** TGCA-STAD from **D)** ACRG repository. Each dot represents a patient. Results are the mean ± SEM. * p < 0.05.

**Figure S2. A)** H&E staining (on left) and IF staining (on right) of LIFR (red) and FGFR4 (green) on non-neoplastic, intestinal type and diffuse type of GC patients derived-mucosa. Kaplan-Meier survival curve shows 5 years survival in GC patients with high expression levels of **A)** FGFR4 and **B)** LIF from our cohort of patients, **C)** FGFR4 and **D)** LIF from STAD-TGCA repository, **E)** FGFR4 and **F)** LIF from ACRG repository.

**Figure S3.** Kaplan-Meier survival curve shows 5 years survival in GC patients with high expression levels of **A)** FGFR4 and **B)** LIF from our cohort of patients, **C)** FGFR4 and **D)** LIF from STAD-TGCA repository, **E)** FGFR4 and **F)** LIF from ACRG repository in intestinal subtype of GC. Kaplan-Meier survival curve in **G)** FGFR4 and **H)** LIF from our cohort of patients, **I)** FGFR4 and **J)** LIF from STAD-TGCA repository, **K)** FGFR4 and **L)** LIF from ACRG repository in diffuse subtype of GC.

**Figure S4.** Relative mRNA expression of **A)** LIFR, **B)** LIF, **C)** FGFR4 and **D)** FGF19 in MKN45, MKN74 and KATO III cells naÏve. Each value is normalized to GAPDH and is expressed relative to those of positive controls, which are arbitrarily set to 1. Results are the mean ± SEM of three samples for group.

**Figure S5**. **IFD docking results and synthetic process**. **A)** Two-dimensional structure of compounds LRI-101, LRI-103 and LRI-201. **B)** IFD docking poses. The ligands of the three complexes, hLIFR-LRI-101, -LRI-103, and -LRI-201, are depicted in green, grey and yellow, respectively. At the same time, the principal amino acid residues implicated in the binding mode are labelled and highlighted in the stick model. All chemical pharmacophore features are highlighted for each complex, and the hydrogen bonds are pictured as dashed lines. **C)** Reagents and Conditions. **a)** (CF3)2CO, H2O2, Na2HPO4, dry CH2Cl2, 0°C, 72%; **b)** 4-chlorophenylMgBr or biphenylMgBr, CuCl, dry THF, 0°C; **c)** (CH3CO)2O, DMAP, pyridine, 65°C, 51 and 45% in two steps, respectively; **d)** acetone cyanohydrin or allylmagnesium bromide, THF dry, 0°C then RT, quantitative, 90%, and 84%, respectively; **e)** HCl 6N, in THF : MeOH 1:1 v/v, 15, 17 and 36% respectively.

**Figure S6. MD analysis of hLIFR-LRI-201 complex**. **A)** 3D and 2D views of the best IFD docking pose and the most representative clusters (c0, c1 and c2) after 200 ns of MD simulation. The ligand and the principal amino acid residues involved in the binding mode are labelled and highlighted in the stick model. All chemical pharmacophore features are highlighted for each complex, and the hydrogen bonds are pictured as dashed lines. **B)** Clusters distribution and ligand root means square deviation (L-RMSD) graphs after 200ns of MD simulations. **C)** Distance hydrogen-bonds analysis during 200 ns of MD simulation between (left) C3-keto and (right) C17-hydroxyl groups with T308 (black) and D283 (red), and N339(black), K332 (red) and T338 (blue), respectively.

**Figure S7. NMR**

**Figure S8.** Gastric organoids were established from healty C57BL6/J mice. Data shown are: **A)** H&E staining of gastric organoid. **B)** IF analysis of E-CADH (green) and LIFR (red) basal expression. Gastric organoids were exposed to LIF (10 ng/ml) alone or in combination with LIF201 (20 µM) for 1 week. **C)** Representative photos of 3D cultures of the three experimental groups. **D)** Number of single cells derived from 3D culture dissociation. Relative mRNA expression of **E)** LIFR and **F)** FGFR4. Results are the mean ± SEM of two samples for group (* represents statistical significance versus NT, and # versus LIF, *p < 0.05).
