# Supplementary material for: The leukemia inhibitory factor regulates fibroblast growth factor receptor 4 transcription in gastric cancer
Source: Cell Oncol (Dordr). 2023 Nov 9;47(2):695–710. doi: 10.1007/s13402-023-00893-8 (PMC11090936; doi:10.1007/s13402-023-00893-8)
Supplement: Supplementary file 10 — Supplementary file10 (DOCX 16 KB) [file 13402_2023_893_MOESM10_ESM.docx]

**Table S1**. Alpha Screen assay results.

| **Compound** | **% inhibition at 50 µM** | **IC_50_ LIFR/LIF** |
| --- | --- | --- |
| LRI-101 | 58.27 | 15.50 ± 2.56 µM |
| LR1-103 | 48.30 | 38.13 ± 3.56 µM |
| LRI-201 | 54.93 | 21.92 ± 2.16 µM |

**Table S2**. Cluster analysis of the hLIFR-LRI-201 complex after 200ns of MD simulation and MM/GBSA ΔG value energy estimation.

| Cluster | % pop | AvgDist^a^ | Stdev | AvgCDist | MMGBSA^b^ (ΔG)^c^ |
| --- | --- | --- | --- | --- | --- |
| c0 | 43 | 0.624 | 0.154 | 1.238 | -42.34 (±1.2) |
| c1 | 38 | 0.574 | 0.154 | 1.353 | -35.18 (±1.4) |
| c2 | 11 | 0.682 | 0.174 | 1.373 | -48.80 (±2.5) |
| c3 | 0.1 | 0.705 | 0.192 | 1.285 | - |
| c4 | 0.0 | 0.555 | 0.160 | 1.391 | - |

^a^AvgDst represents the maximum RMSD in Å from the other member of the cluster; ^b^Calculated with CPPTRAJ module; ^c^ΔG expressed in kcal/mol.
